# Cardiac Magnetic Resonance in Rheumatology to Detect Cardiac Involvement Since Early and Pre-clinical Stages of the Autoimmune Diseases: A Narrative Review

**DOI:** 10.3389/fcvm.2022.870200

**Published:** 2022-07-13

**Authors:** Lilia M. Sierra-Galan, Mona Bhatia, Angel Leovigildo Alberto-Delgado, Javier Madrazo-Shiordia, Carlos Salcido, Bernardo Santoyo, Eduardo Martinez, Maria Elena Soto

**Affiliations:** ^1^Cardiology Department of the Cardiovascular Division of the American British Cowdray Medical Center, Mexico City, Mexico; ^2^Department of Imaging, Fortis Escorts Heart Institute, New Delhi, India; ^3^Cardiology Department of the Central Military Hospital of the Secretary of National Defense, Mexico City, Mexico; ^4^Immunology Department of the National Institute of Cardiology, “Ignacio Chavez”, Mexico City, Mexico

**Keywords:** autoimmune disease, cardiac MRI, fibrosis, late gadolinium enhancement, thrombosis, antiphospholipid, lupus, rheumatoid arthritis

## Abstract

Autoimmune diseases (ADs) encompass multisystem disorders, and cardiovascular involvement is a well-known feature of autoimmune and inflammatory rheumatic conditions. Unfortunately, subclinical and early cardiovascular involvement remains clinically silent and often undetected, despite its well-documented impact on patient management and prognostication with an even more significant effect on severe and future MACE events as the disease progresses. Cardiac magnetic resonance imaging (MRI), today, commands a unique position of supremacy versus its competition in cardiac assessment and is the gold standard for the non-invasive evaluation of cardiac function, structure, morphology, tissue characterization, and flow with the capability of evaluating biventricular function; myocardium for edema, ischemia, fibrosis, infarction; valves for thickening, large masses; pericardial inflammation, pericardial effusions, and tamponade; cardiac cavities for thrombosis; conduction related abnormalities and features of microvascular and large vessel involvement. As precise and early detection of cardiovascular involvement plays a critical role in improving the outcome of rheumatic and autoimmune conditions, our review aims to highlight the evolving role of CMR in systemic lupus erythematosus (SLE), antiphospholipid syndrome (APS), rheumatoid arthritis (RA), systemic sclerosis (SSc), limited sclerosis (LSc), adult-onset Still's disease (AOSD), polymyositis (PM), dermatomyositis (DM), eosinophilic granulomatosis with polyangiitis (EGPA) (formerly Churg-Strauss syndrome), and DRESS syndrome (DS). It draws attention to the need for concerted, systematic global interdisciplinary research to improve future outcomes in autoimmune-related rheumatic conditions with multiorgan, multisystem, and cardiovascular involvement.

## Introduction

Rheumatologists know cardiac involvement among diseases within the spectrum of autoimmunities, such as systemic lupus erythematosus (SLE) or antiphospholipid syndrome (APS). However, other less frequent conditions seen in their daily practice may impact the heart, which is rarely recognized during the subclinical period.

Autoimmune disease (AD) comprises a broad spectrum that affects many levels and structures of the cardiovascular (CV) system. However, some of them, such as the different vasculitides and the spondyloarthropathies, are too extensive to be part of a generic revision since each could be a complete chapter. Therefore, to decide what diseases are included in this review, we conducted a literature search, as shown in the following algorithm that describes the broad spectrum of this group of pathologies beyond the scope of this review.

Different non-invasive CV imaging modalities play an essential role in diagnosing the involvement of the heart in these diseases; however, their diagnostic accuracy varies depending on their pretest probability, the type and severity of involvement, and the local expertise. Therefore, such involvement is not always detected during their early phases (Table 1). Cardiovascular magnetic resonance (CMR) is an imaging modality that could have a relevant role in ADs. However, it is not well recognized and with limited widespread due to different reasons described in this review in more detail.

**Table 1:** CMR value and Pros and Cons compared with other imaging modalities.

**Underlying mechanism of the disease**	**ECG**	**Chest X-ray**	**CT scan**	**Echo**	**CMR**	**Nuclear (SPECT/PET)**
VHD-Libman-Sacks endocarditis	No	No	Yes, if large	Yes	Yes	PET, yes, if large
Pericardial effusion	No—just a very large one		Yes	Yes	Yes	CT aspect of PET, if large
Pericarditis—inflammation	Severe ones	No	No	No	Yes	PET, if severe
Noncalcified constrictive physiology of the pericardium	No	No	No	Yes	Yes	No
RV dysfunction	Late	Late	Late	Yes, but not always	Yes	No
LV dysfunction	Late	Late	Late	Not subclinical	Yes	Late
Myocardial edema	No	No	No	No	Yes	No
Myocardial ischemia	Yes	No	No	Yes, indirectly	Yes	Yes
Myocardial infarction	No smaller ones	No	No	Yes, not small or subendocardial	Yes	Yes, but not small or subendocardial
Myocarditis	No	No	No	No, just large ones	Yes	SPECT—No^*^ PET—Yes
Coronary artery disease	Yes	No	Yes-CCTA	Not directly	Yes	Not directly
Microvascular dysfunction	Yes	No	No	No	Yes	Only PET
Aortic involvement	No	Large ones	No, in subclinical cases	Aortic root, no in subclinical cases	Yes	No
Pulmonary arteries	No	Late	Yes-CTA till distal branching	Main trunk and branches	Yes-till proximal branching	No
Pulmonary hypertension	Late	Late	Late	Yes	Yes	No
Radiation free	Yes	No	No	Yes	Yes	No
Availability	++++	++++	+++	++++	+++	++
Costs	+	+	++	++	++/+++	+++/++++
Claustrophobia	No	No	Yes—very rare	No	Yes	Yes—rare
Renal function contraindication	No	No	Yes	No	No^**^	No
Implantable (metal) devices contraindication	No	No	No	No	No^**^	No
Recommended for routine use in younger people than 65 years old, mainly women—radiation risk	Yes	Yes	No	Yes	Yes	No
Reproducibility	Yes	Yes	Yes	No-operator dependent	Yes	Yes
Prognostic information	No	No	No	Yes	Yes	Yes

* *SPECT had the technique of Gallium-67, currently obsolete due to inherited limitations of the study; Tc^99^m could be helpful in some cases*.

** *Currently, there are no absolute contraindications, contrast media could be used in renal impairment, and MRI scanning is possible with implantable devices. See dedicated literature for details and specifications*.

## CMR Technique

This non-invasive imaging modality is considered the gold standard for many quantitative measurements of cardiovascular disease. Moreover, extensive scientific evidence supports a useful diagnostic tool for different pathologies such as ischemic cardiomyopathy (ischemia and viability), diverse non-ischemic cardiomyopathies, myocarditis, right ventricular disease, and congenital heart disease (1). Up to date, there are constantly updated standards, consensus, and clinical indications (2), which is slowly gaining more and more class I indications in European and American Guidelines (3–20) and position statements (21–26). Currently, the are standard data acquisition (27), guides on how to interpret (28) and structure a comprehensive report (2), and which allows extracting the most information from a CMR scan to maintain its high reproducibility and to feed international registries and databases to gain more clinical indications based on evidence are constantly updating during the past two decades by the Society for Cardiovascular Magnetic Resonance (SCMR).

Unfortunately, nowadays, many referring physicians have little or no training in this technique, provoking an unintended lack of knowledge of CMR general principles, terminology (1), and clinical indications; which allows the global misconception of the recognized cons of the technique to become more robust and sometimes even a myth that limits its proper application on the benefit of the patients.

The SCMR recognizes that several reasons exist for this problem which increases the complexity of selecting appropriate testing for a given clinical situation, such as the complex underlying physics and terminology that are not intuitively understood by the referring physician or non-CMR-expert, along with multiple and even vendor-specific terms uses for the same technique. To help the referring physicians and the non-CMR-expert users, a simplified CMR terminology was officially launched in 2014 by the Society to be used in clinically oriented publications to improve the acceptance and widespread use of CMR in clinical routine (1).

For a general CMR, the study used in clinical routine, the sequences (type of image) are “black-blood CMR” used to study cardiac structure and morphology; by adding a specific fat saturation, the technique is possible to obtain an “edema CMR” image that is extremely useful to determine the presence and location of water (edema) within the myocardium and to delineate the pericardium in cases where it is thicker than normal. If using a specific type of black-blood CMR sequence is also possible to obtain the “iron CMR,” which allows for identifying and quantifying the iron in the heart. For functional analysis, such as all echocardiographic measurements and projections, CMR has the sequences generally called “cine CMR,” which are high-resolution electrophysiology (ECG) gated images with high endocardial border definition, allowing the analysis of cardiac function during the cardiac cycle. These cine sequences are the gold standard for analyzing left and right ventricular functions for which other imaging modalities are compared and validated (29, 30). To these “cine CMR” sequences, it is possible to add the strain techniques by using post-processing tools commercially available, as in echocardiography (Echo) which allows for analyzing the ventricular mechanics. Using a specific sequence in 2D or 4D, we can obtain the “flow CMR” with 2D flow CMR can get the same information as Echo using Doppler techniques. For more complex anatomy, single acquisition with multiples analysis, without the limitation of patient cooperation currently 4D flow CMR emerged as a valuable option, allowing even the wall stress shear analysis that is fundamental in arterial wall diseases (31).

With the administration of intravenous contrast media, which is a paramagnetic agent (gadolinium), currently approved to be used even in patients with renal dysfunction without the risk of systemic nephrogenic fibrosis development (32), it is possible to obtain the “perfusion CMR” by analyzing the first pass of the contrast material into the vascular system first into the cardiac chambers and then into the myocardium allowing the identification and quantification of normal and abnormal perfusion in areas of myocardial ischemia or necrosis. The intravenous administration of the contrast media can be used to obtain a high-resolution magnetic resonance angiography from the supra-aortic vessels down to the aortic bifurcation within the same cardiac MRI study. By waiting 7–10 mins after gadolinium administration, when other images can be obtained, the “late gadolinium enhancement (LGE) CMR” images are broadly recognized ones showing myocardial necrosis and fibrosis. Recently, the addition of mapping techniques during this same scan time acquisition, the T1 and T2 mapping with the process of the extracellular volume map allows the identification of the diffuse process of inflammation, edema, and fibrosis; which is essential for the ADs mainly in early stages before becoming clinically evident (1).

Since up-to-date CMR is not included in the Clinical Guidelines for evaluating ADs with CV involvement, the following case description will serve as the preamble for the rest of the manuscript.

A young woman, 34 years old, was recently diagnosed with RA and suddenly started with signs and symptoms of heart failure (HF), which was reviewed by her Rheumatologist, who considered that she would need to see a Cardiologist. During her consultation with an expert cardiologist, she was diagnosed using ECG, laboratory tests, and Echo as having mild left ventricular dysfunction with moderate pericardial effusion with no constrictive physiology; and she was started on proper maximum medical treatment for this condition. However, after two weeks of no improvement and being re-evaluated by her Rheumatologist, she was asked to take a second cardiology opinion with a cardiologist expert in ADs and advanced cardiovascular imaging. After being evaluated by the second cardiologist and using only the same information she had for her first cardiologist, she was recommended for an oriented autoimmune cardiac MRI protocol, where she was identified as having a normal left ventricular function, moderate pericardial effusion, unresponsive to the standard treatment and additional three key findings, the presence of amyloidosis probable secondary and related to the RA, an inflammatory component of the pericardial disease and the involvement of valvular and vascular structures. As a result, her Rheumatologist modified the medical treatment to a more aggressive immunomodulatory agent with an excellent immediate response (33).

This case nicely illustrates and explains the motivation of the title and the purpose of this manuscript to review the pathophysiology of cardiac involvement and the cardiovascular magnetic resonance (CMR) findings of ADs.

**Figure 1:**
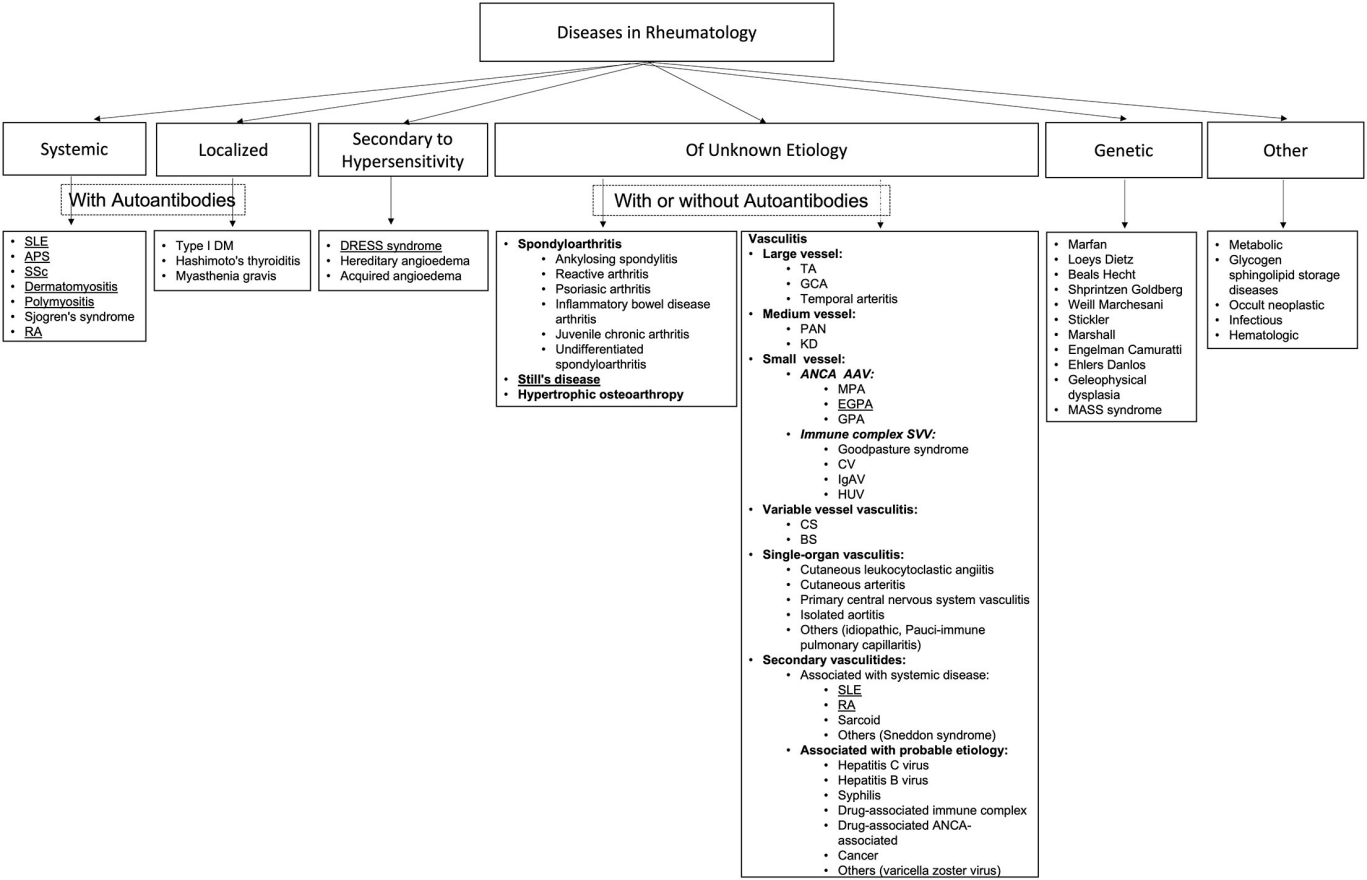
Algorithm of representative rheumatological disorders. This figure shows an algorithm created using the published literature that includes most rheumatological disorders. The algorithm is divided into six big categories as systemic and localized (both with autoantibodies), one group secondary to hypersensitivity, another large group of diseases of unknown etiology (with or without autoantibodies) which mainly involve spondyloarthropathies and vasculitides (34), one group of genetic origin and finally a group of other sub-groups of disorders such as those related to neoplasias, infectious and metabolic diseases. By looking at the algorithm, it is comprehensive that one general review paper could not go into detail for each component. Therefore, we chose to describe the most frequent diseases with known cardiovascular involvement from the systemic with autoantibodies groups, such as those underlined ones—one representative illness of the group of spondyloarthropathies and one from the group of vasculitides. Finally, one infrequent, maybe underdiagnosed disease such as the DRESS syndrome from the group of diseases secondary to hypersensitivity. SLE, systemic lupus erythematosus; APS, antiphospholipid syndrome; SSc, systemic sclerosis; RA, rheumatoid arthritis; DM, diabetes mellitus; TA, Takayasu arteritis; GCA, giant cell arteritis; PAN, polyarteritis nodosa; KD, Kawasaki disease; ANCA AAV, Antineutrophil cytoplasmic antibody (ANCA)-associated vasculitis (AVV); MPA, microscopic polyangiitis; EGPA, eosinophilic granulomatosis with polyangiitis (formerly Churg-Strauss syndrome); GPA, granulomaotsis with polyangiitis (Wegener's); SVV, small vessel vasculitis; CV, cryoglobulinemic vasculitis; IgAV, IgA vasculitis (Henoch-Schönlein); HUV, hypocomplementemic urticarial vasculitis (anti-C1q vasculitis); CS, Cogan's syndrome; BS, Behcet's syndrome.

There are many different diseases in the field of Rheumatology, including those considered systemic or localized with autoantibodies, those secondary to hypersensitivity, and others with unknown etiology with or without autoantibodies that involve spondyloarthropathies and vasculitides (34) those of genetic origin and some other miscellaneous as are shown in the algorithm of Figure 1.

Based on the literature research and the algorithm, we decided to include in this review the diseases that are of autoimmune origin with systemic presentation and known CV involvement and high prevalence, such as systemic lupus erythematosus (SLE), antiphospholipid syndrome (APS), rheumatoid arthritis (RA), systemic sclerosis (SSc), polymyositis (PM), dermatomyositis (DM); one disease from the group of spondyloarthropathies, the adult-onset Still's a disease (AOSD), one disease from the group of vasculitides, the eosinophilic granulomatosis with polyangiitis (EGPA) (formerly Churg-Strauss syndrome), and finally, one rare condition secondary to hypersensitivity, the DRESS syndrome (DS) as an example of the complexity of the field, the high risk of cardiovascular involvement in specific clinical presentations that are not routinely recognized as autoimmune that could be even underdiagnosed and reported.

Spondyloarthropathies and vasculitides (34) are too extensive to be part of a general revision such as this one and deserve an entire chapter for each one.

We theorize the potential role of CMR in the screening, diagnosis, prognosis, management guidance, and follow-up of response to treatment supported by current scientific evidence.

### Systemic Lupus Erythematosus

#### General Description

**Figure 2:**
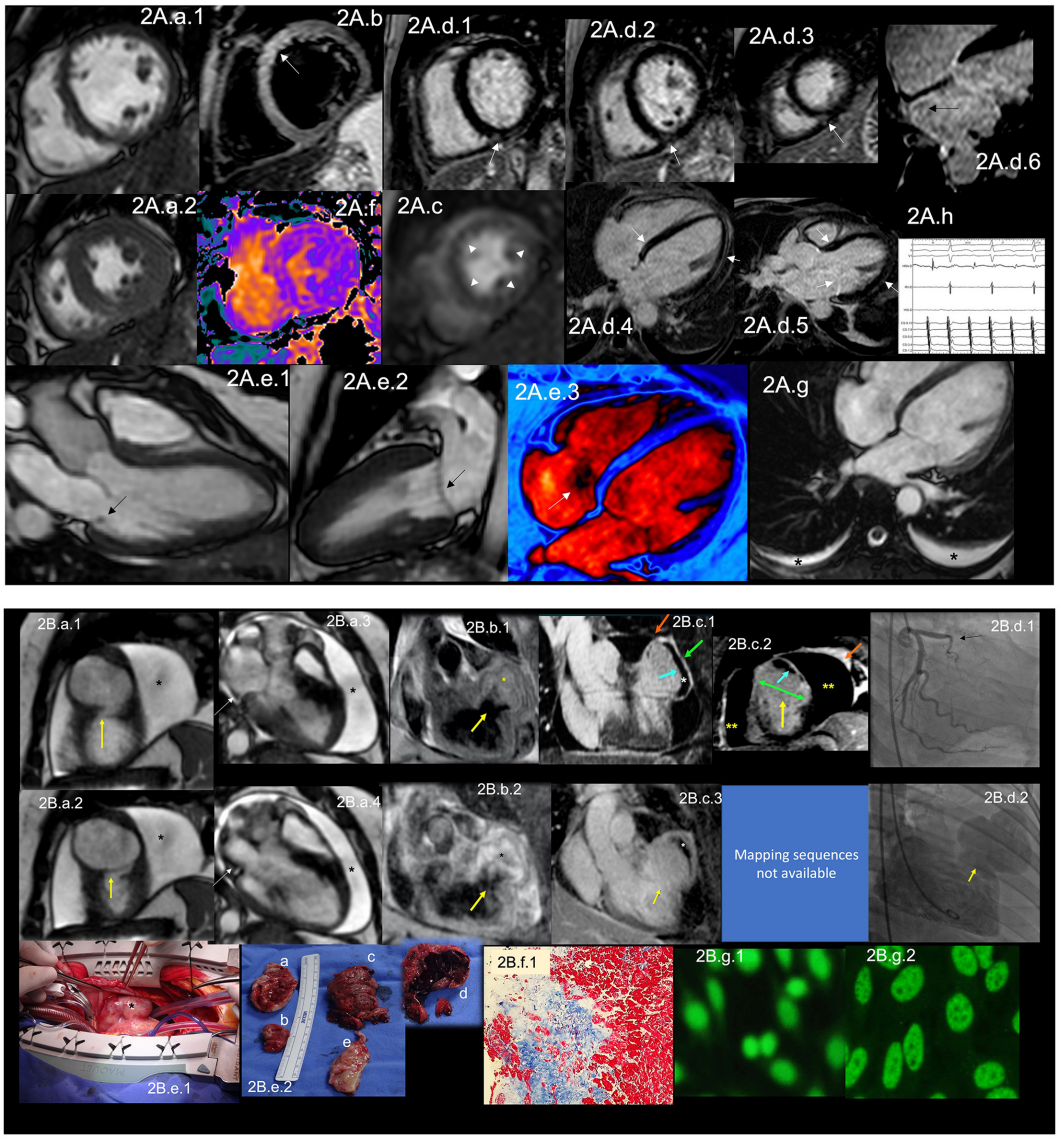
**(A)** CMR findings in SLE—**(A)** from an arrhythmic perspective. A panel figure demonstrates the array of findings from a comprehensive CMR study in SLE from an arrhythmia perspective. **(A.a)** Shows normal global and regional right and left ventricular functions in SSFP sequence still cine images in short-axis views at the mid-ventricular level in end-diastole **(A.a.1)** and end-systole **(A.a.2)** LVEF 66% and RVEF 63%. **(A.b)** T2-W STIR sequence in short-axis view projection at a mid-ventricular level demonstrating mildly increased signal intensity in the anterior, anteroseptal, and inferoseptal segments (arrow) with a myocardial/skeletal muscle ratio of 2.8 and **(A.f)** a color-coded T2 mapping (native T2 value of 48 ms) consistent with myocardial edema. **(A.c)** Stress CMR with FPP T1-W sequence with adenosine infusion at 140 mcg/kg/min over 6 minutes shows a subendocardial ring perfusion defect (arrowheads) consistent with subendocardial ischemia due to endothelial dysfunction and probably microvascular disease (no obstructive coronary artery disease in CCTA). **(A.d.1)** LGE PSIR sequence. **(A.d.1–3)** short-axis views at basal **(A.d.1)**, mid **(A.d.2)** and apical **(A.d.3)** levels, and long-axis views in 4-chambers **(A.d.4,6)** and 3-chambers **(A.d.5)** projections. The arrows in these images show areas of focal fibrosis in the subepicardium in the inferior segments in **(A.d.1–3)** from the base to the apex. **(A.d.4,5)** shows areas of midventricular LGE in basal anteroseptal and inferoseptal segments (arrows) and subepicardial enhancement in mid and apical inferolateral segments (arrow). **(A.d.6)** A zoomed image of the 4-chambers view showing LGE suggestive of LA fibrosis (arrow). **(A.h)** A polygraphic trace of an EP study showing atrial fibrillation successfully ablated. **(A.e.1–3)** SSFP sequence still cine images in long-axis views, showing in a 3-chambers view an isointense, small nodule, in the ventricular side of the mitral valve suggestive of Libman-Sacks endocarditis (arrow) (confirmed by echo) **(A.e.1)**, in a 2-chambers view, a mildly thickened mitral valve **(A.e.2)** and in a color-coded 4-chambers **(A.e.3)** the presence of tricuspid regurgitation (arrow). **(A.g)** An SSFP sequence still cine images in true axial view showing bilateral pleural effusion (asterisks). SLE, systemic lupus erythematosus; CMR, cardiovascular magnetic resonance; SSFP, steady-state free precession; LVEF%, left ventricular ejection fraction; RVEF%, right ventricular ejection fraction; T2-W STIR, T2-weighted short-tau inversion recovery; FPP, first-pass perfusion; T1-W, T1-weighted; CCTA, invasive coronary angiography; LGE, late gadolinium enhancement; PSIR, phase-sensitive inversion recovery; LA, contrast enhancement magnetic resonance angiography; EP, electrophysiology. **(B)** CMR findings in SLE—**(B)**, from a thrombotic perspective. A panel figure demonstrates the array of findings from a comprehensive CMR study in a 1.5 T scanner of SLE from a coronary thrombosis perspective. **(B.a.1–4)** SSFP sequence still cine images in short-axis views at mid to apical ventricular level in end-diastole **(A.a.1)** and end-systole **(A.a.2)** and long-axis 3-chambers view, in end-diastole (**B.a.3**) and end-systole **(B.a.4)** that shows in **(A.a.1)** the loss of continuity of the LV anterior segment (yellow arrow), with normal thickening of the remaining segments **(B.a.2)** surrounded by a large pericardial effusion (black asterixis), that exhibits hemodynamic compromise as the diastolic collapse of the LA [yellow arrow in **(B.a.3,4)**]. LVEF 42% and RVEF 65%. **(B.b,c)** Show the tissue characterization findings. **(B.b.1)** T1-W sequence in long-axis view demonstrating a wide-necked outpouching of the LV anterior wall with the apparent loss of myocardial continuity with a thinned out, fibrosed muscle surrounding the cavity consistent with true aneurysm (yellow arrow) with tissue within the aneurysmal cavity of two different intensities (yellow asterisks) suggestive of thrombus. **(B.b.2)** T2-W STIR sequence in long-axis view confirming the findings of T1-W sequences (yellow arrow) with the evident different signal intensity of the tissue components inside the aneurysmal cavity (black asterixis) suggestive of two varying ages of the thrombus, recent and old, and slow-flowing blood. **(B.c)** LGE PSIR sequence. **(B.d.1)** Long-axis 3-chambers view showing loss of myocardial continuity, a large cavity surrounded by scarred myocardium (green arrow) with a large thrombus on its endocardial aspect (white asterisks), pericardial enhancement (orange arrow), and a large pericardial effusion (yellow double asterisks) that are confirmed on the corresponding short-axis view **(B.c.2)**. **(B.c.3)** A long TI LGE-PSIR long-axis 3-chambers view confirming previous data and showing the new thrombus component (yellow asterisks). **(B.d.1)** An invasive angiography demonstrating a total occlusion of the proximal LAD (arrow) and the invasive ventriculography **(B.d.2)** Showing a large leak of contrast media at an anterior mid-ventricular level impossible to differentiate aneurysm from pseudoaneurysm. **(B.e.1)** Open heart surgery showing the intact LV wall covered by the pericardium consistent with a true aneurysm (arrow) which was successfully resected **(B.e.2)**, and surgery confirmed CMR findings, a ventricular aneurysm **(B.e.2.a,b)**, a transmural scar **(B.e.2.e)** and a large thrombus **(B.e.2.c)** composed of two different aged thrombi **(B.e.2.d)**. H.E. stain histology confirmed the presence of a large scar with no evidence of atherosclerosis **(B.f.1)**. Based on the inflammatory component of the pericardium, suspicion of autoimmune instead of atherosclerotic etiology was suspected and confirmed by the finding of ANA with a homogeneous pattern in the pericardial effusion **(B.g.1)** and peripheral blood of a fine speckled pattern **(B.g.2)**. SLE, systemic lupus erythematosus; CMR, cardiovascular magnetic resonance; SSFP, steady-state free precession; LVEF%, left ventricular ejection fraction; RVEF%, right ventricular ejection fraction; LV, left ventricle; LA, left atrium; T2-W STIR, T2-weighted short-tau inversion recovery; FPP, first-pass perfusion; T1-W, T1-weighted; ICA, invasive coronary angiography; LGE, late gadolinium enhancement; PSIR, phase-sensitive inversion recovery; TI, time to inversion; LAD, left anterior descending artery; H.E., hematoxylin and eosin.

Since 1924, when Libman and Sacks first published the association of aseptic vegetations and SLE (35), we have known specific manifestations involving the CV system in the context of ADs. CV involvement in SLE occurs at pericardial, valvular, and myocardial levels (Figures 2A,B). CVD is the leading cause of death in SLE patients (36–39). It is estimated that SLE patients have a two-fold risk of developing MI or stroke compared to the general population (38). Premenopausal women with SLE have a 50-fold more risk of MI than healthy matched controls (40, 41).

Recently, the 2019 update of the EULAR recommendations (42) for the management of SLE state that patients should undergo regular assessment for traditional and disease-related risk factors for CVD, including persistently active disease, increased disease duration, medium/high titers of antiphospholipid antibodies, renal involvement, and chronic use of glucocorticoids; and based on the CV risk profile patients may be candidates for low dose aspirin or lipid-lowering therapies (42). Unfortunately, classic risk scores for evaluating CVD can underestimate risk in SLE patients since they are primarily young women (43). In this matter, Petri et al. developed a formula to evaluate CV risk in SLE patients based on disease-related risk factors (44). Elevated CV risk in SLE patients at younger ages, particularly with disease-related risk factors, can prompt an evaluation of CVD risk in this population.

#### Clinical Manifestations of CV Involvement and Pathogenesis of Cardiac Manifestations

The most common CV manifestation is pericarditis presenting with pericardial effusion, which can occur in 11–54% of cases (39) during the disease (Figure 2A.a.1,2,2B.a.1–4,c.1–3). Even though it is the most common CV manifestation, cardiac tamponade rarely develops. It is associated with positive antinuclear antibodies (ANA), fever, and chest pain, just as acute viral pericarditis, so excluding other causes of pericardial disease in these patients is mandatory. Still, in disease activity, pericarditis is rarely due to other etiologies (39).

Valvular heart disease in SLE involves more than Libman-Sacks endocarditis (Figure 2A.e.1). Valve thickening (Figure 2A.e.2) and valvular dysfunction (Figure 2A.e.3) can also occur (45). In a study, Vivero et al. included 211 patients with SLE. Of those, 53 had significant valvular involvement; however, they found no valve vegetations in any of these patients (46). In a meta-analysis by Hussain et al., they included 2556 SLE patients. The most commonly involved was the mitral valve, with mitral regurgitation being the most common valve disease. Other lesions were mitral stenosis, tricuspid regurgitation (Figure 2A.e.3), and aortic regurgitation (47). In the same study, the authors found that compared to control subjects, SLE patients had an increased risk of developing valve disease, the highest risk being valvular thickening (RR 6.99, CI 3.64–13.44) and valvular vegetations (RR 7.73, CI 3.09–19.3) (47). VHD has been linked to high titers of antiphospholipid antibodies (aPL) (48, 49).

Specific manifestations can cause HF in SLE, which has been reported with a prevalence of 1–10% in SLE (47, 50). MI and coronary artery disease (CAD) are the leading causes of CVD in these patients (Figure 2B), besides being significant risk factors for the development of HF (50). Treatment of SLE with disease modifier drugs such as corticosteroids or hydroxychloroquine is further associated with increased CV risk (35) related to dose-dependent cardiotoxicity and resultant manifestations of restrictive cardiomyopathy (51). Dhakal et al. described risk factors associated with HF development in SLE. Traditional risk factors such as smoking, obesity, hypertension, CAD, advanced age, and male sex play a role in HF development, but also there are disease-specific risk factors such as left ventricular hypertrophy, described in as much as 20% of cases, myocarditis, chronic kidney disease, and vasculitis, among others (50). Primary myocardial involvement manifested as myocarditis affects ~3–9% of SLE patients (Figure 2A.b,d.1–5,f), while African-Americans are at higher risk (39).

Regarding the pathophysiology of CV manifestations in SLE, there is a role of immune complexes in CV manifestations of SLE. In pericarditis, for example, granular depositions of immunoglobulins and C3 complement have been found in pericardial tissue, and neutrophil predominance in pericardial fluid exudate, ANA (Figure 2B.g.1,2), and other autoantibodies can be found (39).

Even though autoimmunity is the hallmark of the disease, and immune complexes are presumed to be related to the CV manifestations of SLE, the exact pathophysiology behind them is still only somewhat understood (52).

Endothelial dysfunction (Figure 2A.c) is accepted as the central hypothesis of CVD, especially CAD, in SLE patients. The mechanism by which this occurs is complex. Roughly, endothelial dysfunction is caused by several pathways, including expression of vascular cell adhesion molecules, which correlates with higher coronary artery calcium scores (53), activation of type I interferon and IFN-α, which inhibit eNOS expression at protein and mRNA levels which impair insulin-mediated nitric oxide production in endothelial cells (54). Cellular mediators for endothelial dysfunction involve low-density granulocytes which form neutrophil extracellular traps that promote vascular leakage and activate the B-catenin signaling pathway (38). Conversely, T cells play a proatherogenic role through their migration to the arterial wall (55). Mercurio et al. reported increased radial artery stiffness, increased aortic pulse pressure, and its correlation with some inflammatory markers such as C-reactive protein (CRP) and erythrocyte sedimentation rate (ESR), but not with disease activity in 43 SLE patients. The authors concluded that inflammation is the primary determinant of CV complications in SLE (56).

MI is mainly due to coronary atherosclerosis, but it might be due to acute *in situ* coronary thrombosis when there is an association with aPL (57). Arterial thrombosis manifests as coronary thrombosis in 23% (58) (Figure 2B.d.1,2). It has been described that MI secondary to coronary *in situ* thrombosis can be the first manifestation of SLE and APS which can be complicated by catastrophic APS (57) (Figure 2B). Treatment for STEMI in these patients must be as stipulated in current guidelines.

In patients with lupus myocarditis, the typical findings on endomyocardial biopsy are mononuclear cell infiltration, perivascular inflammation, and cardiomyocyte necrosis with granular immunoglobulins and complement deposition, which support the role of immune complexes (50).

Although it is not the most common manifestation of SLE, serositis is one of the American College of Rheumatology (ACR) classification criteria to define the disease (59). Serositis can manifest as pleurisy (Figure 2A.g) with pleural effusion, which occurs in 15–34% (60), or as pericarditis with pericardial effusion, which is the most common CV manifestation and can occur in 11–54% of patients with SLE (39, 60), during the disease. A study of the Hopkins Lupus Cohort evaluated predictors of pleurisy and pericarditis among different populations. They found that African-American ethnicity, male gender, and serological markers such as ESR, Anti-DNA, and low C3/C4 were strong predictors for serositis in the form of pleurisy and/or pericarditis (60).

#### CMR Role

Autopsy studies in patients with SLE show cardiac involvement in up to 40% of patients, of which only 10% were clinically diagnosed (40). Even though echocardiography is the leading non-invasive imaging modality in the initial evaluation of CVD, it might not be the appropriate initial modality in SLE patients since it is not ideal for tissue characterization. Mavrogeni et al. analyzed patients referred to evaluate typical and atypical cardiac symptoms with normal Echo and connective tissue diseases. They found that 25.2% of these patients had myocardial fibrosis. When the percentage of LV mass with late gadolinium enhancement (LGE) exceeded 5%, they were at increased risk of future cardiac events (61). Burkard et al. evaluated 30 SLE patients, mainly female, with no history of CAD and found that 43% (*n* = 13) had abnormal CMR; the main findings were the presence of LGE (Figure 2A.d.1–5,B.c.1–3), stress perfusion abnormalities (Figure 2A.c), and pericardial effusion (Figure 2B) (40). This reinforces the need for early CMR evaluation in these patients mainly for (1) detection of acute disease that cannot be diagnosed with Echo (e.g., arterial wall inflammation, asymptomatic myocardial involvement). (2) Evaluation of CAD with CMR perfusion techniques in patients with high CV risk-stratified with novel tools. (3) Evaluation of pericardial tissue. (4) Evaluation of valve involvement and (5) early initiation of cardiac protective treatment in patients with detected myocardial involvement, considered a risk factor for HF in these patients (62–64). See Table 2 for detailed CMR offerings to SLE and Table 1 for pros and con over other imaging modalities.

**Table 2:** CMR offerings to the SLE.

**Cardiac manifestation**	**CMR utility**	**Sequences**
**What has CMR to offer in systemic lupus erythematosus?**		
Valvular Heart Disease	Assessment of hemodynamic significance. Reproducible follow-up.	Cine—SSFP, cine-FGE, PhC, 4D-flow
Pericarditis/Pericardial Effusion	Identification Assessment of severity and hemodynamic significance, Detection of inflammation. Detection of constriction.	Cine—SSFP Free-breathing real-time cine T1-W, T2-W STIR LGE-PSIR
RV dysfunction	The gold standard for RV function	Cine—SSFP Strain
LV dysfunction	The gold standard for LV function	Cine—SSFP Strain, diffusion tensor
Myocarditis	Identification and quantification. Follow up free of radiation.	T2-W T1, T2-mapping, ECV LGE-PSIR
Vascular involvement	Coronary arteries—atherosclerosis, thrombosis, and vasospasm.	Cine SSFP, Stress FPP LGE-PSIR, MR coronary angiography
	Great vessels—Aortic involvement	Cine—SSFP, cine-FGE 4D-flow, MRA
	Microvascular dysfunction	Stress FPP LGE-PSIR

*SSFP, steady-state free precession; FGE, fast gradient echo; PhC, phase contrast; T2-W, T2-weighted; STIR, short tau inversion recovery; LGE, late gadolinium enhancement; PSIR, Phase-sensitive inversion recovery; MRA, magnetic resonance angiography; ECV, extracellular volume; FPP, first-pass perfusion; MR, magnetic resonance*.

### Antiphospholipid Syndrome

#### General Description

Antiphospholipid antibody syndrome is characterized by thrombotic events, pregnancy-related comorbidities, or a series of non-thrombotic symptoms. Thrombotic events are arterial, venous, and/or in the microvasculature. This syndrome could affect the brain, lungs, extremities, and the heart. It is called a catastrophic antiphospholipid syndrome, which involves multiple organs (65). An obstetric form is characterized by three or more consecutive spontaneous pregnancy losses at less than ten weeks of gestation, intrauterine growth restriction, and/or severe preeclampsia. However, other non-thrombotic presentations include valvular disease, livedo reticularis, kidney dysfunction, thrombocytopenia, hemolytic anemia, and cognitive dysfunction (66).

The antiphospholipid antibodies bind the beta-2-glycoprotein I, which is strongly linked to lipid surfaces and favors prothrombotic states by increasing cell adhesion of the E-selectin and tissue factor. It reduces the activity of the C-reactive protein, and the complement is activated. At platelets favors the expression of glycoprotein IIb/IIIa and the activation of neutrophils by generating NETosis and increasing the production of IL-8. Monocytes express higher levels of tissue factor. Recently, increased stimulation of the intracellular mTOR pathway involved in microvascular damage has been described (67).

#### Clinical Manifestations of CV Involvement and Pathogenesis of Cardiac Manifestations

**Figure 3:**
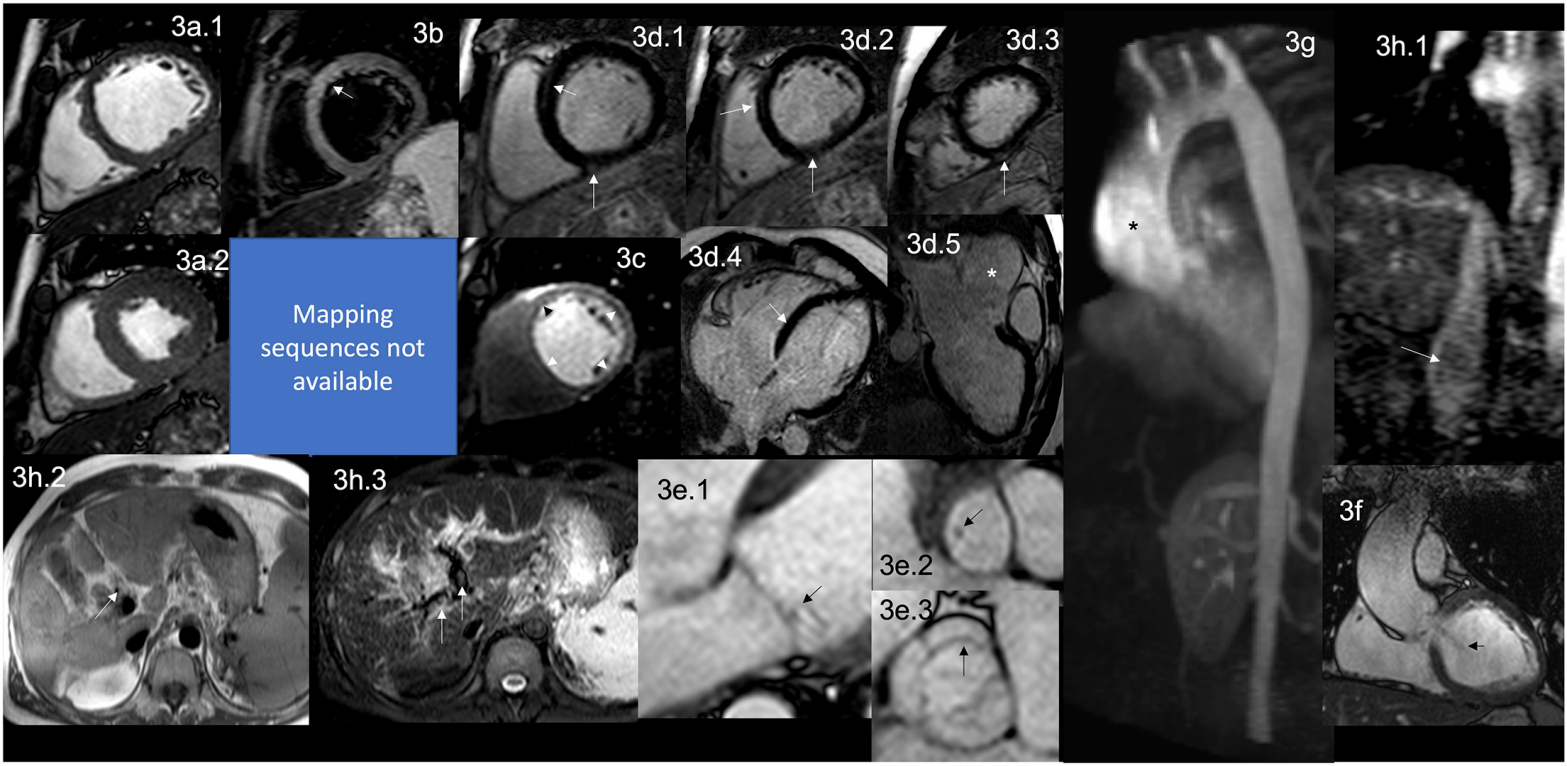
CMR findings in APS. A figure demonstrates the array of findings from a comprehensive CMR study at a 1.5 Tesla scanner of APS. **(a)** SSFP sequence still cine images in short-axis view at the mid-ventricular level in end-diastole **(a.1)** and end-systole **(a.2)** show normal global and regional right and left ventricular functions, LVEF 63% and RVEF 56%. **(b)** T2-W STIR sequence in short-axis view at a mid-ventricular level shows mildly increased signal intensity in the anterior and anteroseptal segments (arrow) with a myocardial/skeletal muscle ratio of 3.6 suggestive of myocardial edema. **(c)** Stress CMR with FPP T1-W sequence with adenosine infusion at 140 mcg/kg/min over 6 mins showing a subendocardial circumferential perfusion defect (arrowheads) consistent with subendocardial ischemia due to endothelial dysfunction and probably to a microvascular disease (no obstructive coronary artery disease demonstrated in ICA). **(d)** LGE PSIR sequence. **(d.1–3)** short-axis views at basal **(d.1)**, mid **(d.2)**, and apical **(d.3)** levels, and long-axis views in 4-chambers **(d.4)** and 3-chambers **(d.5)** projections. The arrows in these images demonstrate areas of focal fibrosis as mid-wall LGE in the anteroseptal segment in **(d.1,d.2,d.4)**; and in the subepicardium of the inferior segment from the base to the apex. **(d.5)** Shows aortic root and ascending aorta dilatation confirmed by the CE-MRA in **(g)** and the coronal view in SSFP still cine image **(f)**, where it also demonstrates the presence of aortic regurgitation (arrow). **(e)** Zoomed still cine images in SSFP sequence of the mitral valve **(e.1)** and a fused asymmetric bicuspid aortic valve **(e.3)** Showing in a 4-chambers view a small isointense nodule in the ventricular side of the mitral valve, suggestive of Libman-Sacks endocarditis (arrow) and in the LVOT [arrow in **(e.2)**] (confirmed by echo), and mild thickening of the fused coronary bicuspid cusp [arrow in **(e.3)**]. Additionally, images **(h.1–3)** Show a thrombus within the portal vein and its main branches; in **(h.1)**, a venous phase of the CE-MRA shows a contrast defect within the portal vein (arrow) consistent with a thrombus. T1-W axial view of the liver **(h.2)** shows a hyperintense structure within the portal system (arrow), and a T2-W STIR view of the liver **(h.3)** shows hypointense masses within the main portal branches (arrow) consistent with thrombus. CMR, cardiovascular magnetic resonance; SSFP, steady-state free precession; LVEF%, left ventricular ejection fraction; RVEF%, right ventricular ejection fraction; T2-W STIR, T2-weighted short-tau inversion recovery; FPP, first-pass perfusion; T1-W, T1-weighted; ICA, invasive coronary angiography; LGE, late gadolinium enhancement; PSIR, phase-sensitive inversion recovery; CE-MRA, contrast enhancement magnetic resonance angiography; LVOT, left ventricular outflow tract.

The pathophysiological abnormalities in APS include vascular thrombosis of large and/or small venous and arterial systems (68). The cardiac involvement presents as valvular heart disease (VHD), intracardiac thrombosis, pulmonary hypertension (PH), right and/or left ventricular dysfunction (Figure 3a.1–2), ischemic heart disease due to involvement of the large epicardial coronary arteries or the microvascular circulation. Thrombosis is less frequent than the VHD but contributes to PH, microvascular (68), or even thrombosis of the epicardial coronary arteries.

It is well described that APS affects the heart valves in approximately 30% of the patients (68). Recently, the association of Libman-Sacks endocarditis with primary APS, and not only when associated with SLE, have been made and demonstrated that it is due to thrombosis and thickening of the valves with the formation of sterile fibro-fibrinous vegetations of the mitral and/or aortic valves on their endocardial surfaces (68–71) (Figure 3e.1–3).

The typical functional abnormality is non-hemodynamically significant mitral and aortic regurgitation, the most frequently affected mitral valve. This valvular involvement is reported as persistent or even progressive over time regardless of the anticoagulant or antiplatelet therapy. Clinically, most patients remain asymptomatic for a long time, but eventually, they will require surgical treatment (68).

Intracardiac thrombosis is rarely reported (72); it produces masses in all four cavities (73–76), and even recurrent masses (77); usually adherent to the endocardial surface (74). These thrombotic masses mimic myxomas (73) or other tumors, even primary ones, by Echo (72). In addition to the presence of cardiac masses, the complication of frequent peripheral embolisms, particularly in the brain, is reported (72).

Many patients without the full criteria for APS or SLE have a stroke or this finding as to the initial manifestation, and since the initial diagnostic workup often does not include CMR; these patients end up at surgery without proper diagnosis and anticoagulation therapy until the histopathological result confirms the presence of thrombotic material suggesting the diagnosis (72–76). Additionally, the myocardial ischemia (see below) causes an ischemic subendocardium which in turn acts as a trigger for thrombus formation, especially if it coexists with left ventricular (LV) dysfunction (see below) (68).

PH is a rare but life-threatening condition in APS with or without SLE (78). The definition of PH is done by Echo based on estimated hemodynamic parameters (TTE) and has to be confirmed by right heart catheterization (RHC) (78). The underlying pathophysiological mechanism involves large vessel and microvascular thrombosis, recurrent pulmonary embolism, and endothelial remodeling (78–80). PH development in APS undergoes progressive worsening unless proper diagnosis and treatment, including anticoagulation, are established. A pulmonary endarterectomy is an option (11, 14), but it is a high-risk procedure with good results only in highly experienced centers (79–81), and it has an increased risk of thrombotic complications (78, 80, 81).

Right ventricular (RV) subclinical dysfunction is more prevalent in APS with or without SLE, and it negatively correlates with the serum levels of anticardiolipin antibodies (ACA) (82). The suggested mechanisms are the underlying inflammation that produces subclinical vasculitis, myocarditis, or vascular stiffening, resulting in ventricular remodeling (83). Once PH has been established, RV dysfunction becomes clinical. However, it could also become life-threatening depending on its severity, the speed of installation, the proper diagnosis, treatment, and other comorbidities.

LV dysfunction is a rare presentation (84, 85) and the suspected mechanism identified in the autopsy is a widespread thrombosis of the intramyocardial arteries and arterioles (86, 87), which one can assume leads initially to microvascular dysfunction, then microinfarctions of the myocardium generating small scars and fibrosis and finally compromising the ventricular function. Myocardial edema (Figure 3b) has (88) also been described as catastrophic (89).

It is infrequent that APS patients develop dilated cardiomyopathy (90). However, a significant correlation exists between high titers of ACA IgM and HF (91).

Ischemic heart disease is generally caused by microvascular endothelial dysfunction (Figure 3c) with or without associated micro thrombosis (86) and minimal inflammation (92).

Myocardial infarction (MI) is due to thrombosis or vasospasm (68) of the large epicardial coronary arteries. MI could be the first manifestation of the disease (93, 94).

#### CMR Role

CMR has identified a high prevalence of myocardial scar (95), diffuse fibrosis (88) (Figure 3d.1–4), and endomyocardial fibrosis (96–98).

As mentioned before, APS can have many cardiovascular manifestations. Therefore, the “APS Task Force” has taken valvular heart disease related to APS as one of the “extra criteria” for its diagnosis (99). Even though echocardiography is usually the first non-invasive imaging technique for evaluating APS-related VHD, CMR has certain advantages, such as tissue characterization, LGE for detecting myocardial fibrosis, and adequate VHD evaluation.

Sacré et al. evaluated 27 consecutive patients with established diagnosis of APS matched with 81 patients without known cardiovascular disease and who developed CMR. LGE was present in 29.6% of APS patients, with a typical ischemic pattern in 11.1%. Remarkably, myocardial scarring had no electrocardiographic nor Echo evidence. Authors concluded that CMR helps search for myocardial ischemia and myocardial fibrosis in APS patients even if they are asymptomatic (95) as CMR can detect scar in as little as 1 cm^3^ of tissue.

In patients with different comorbidities and the coexistence of more than one AD, CMR can help to differentiate by dissecting the components and timing of each one, orienting major procedures such as surgical valve replacements in high-risk patients with specific strategies to lower their risk as much as possible (100).

Since VHD is the most frequent CV manifestation in APS but also myocardial fibrosis, silent ischemia, potential HF, and the presence of thrombus in different sizes might be present (68), CMR might be the non-invasive cardiovascular image of choice in these populations for correct and prompt evaluation of all of these manifestations in APS patients.

Echo helps detect many of the CV involvement in APS. It has many advantages, such as broad availability and the possibility of performing the study on the patient's bed and in hemodynamically unstable ones. As a single modality, it is cheaper. However, it cannot detect the earliest involvement related to tissue characterization such as minor myocardial edema, inflammation, or diffuse fibrosis before it can modify global function or even more sensitive techniques such as strain. CMR can identify in a single study the involvement of different components of the CV system, including great vessels, lungs (where are grossly affected by its vasculature), and stress perfusion with high reproducibility allowing the patient to be its control in the future. It would be necessary to gather information from different imaging modalities such as echo, CT, nuclear, etc., impacting more tests, radiation exposure, and costs (101).

See Table 3 for detailed CMR offerings to APS and Table 1 for pros and cons over other imaging modalities.

**Table 3:** CMR offerings to the APS.

**Pathophysiology**	**CMR offer**	**Sequences**
**What has CMR to offer in antiphospholipid syndrome?**		
VHD: Libman-Sacks endocarditis	Assessment of hemodynamic significance. Reproducible follow-up.	Cine—SSFP, cine-FGE PhC, 4D-flow
Intracardiac thrombosis	High resolution. Location. Tissue characterization. Assessment of hemodynamic significance. Reproducible follow-up, free of radiation.	Cine—SSFP T1-W, T2-W LGE Early LGE Long TI LGE
Pulmonary hypertension	The severity and hemodynamic significance. Orientation to the potential etiology.	Cine—SSFP Real-time cine 4D-flow MRA
RV dysfunction	The gold standard for RV function	Cine—SSFP Strain
LV dysfunction	The gold standard for LV function	Cine—SSFP Strain, diffusion tensor
Myocardial edema	Identification and quantification Follow up	T2-W, T2-W-STIR T2-mapping
Myocardial ischemia	For large epicardial arteries	Stress FPP
	For microvascular circulation	
Myocardial infarction	Identification, location, follow-up free of radiation, prognostic information: • Focal scar • Diffuse fibrosis^*^ • Endomyocardial fibrosis^*^	LGE LGE, T1 mapping, ECV LGE

* *Can also be related to other processes such as myocardial ischemia, pulmonary hypertension, or dilated cardiomyopathy*.

*VHD, valvular heart disease; SSFP, steady-state free precession; FGE, fast gradient echo; PhC, Phase contrast; T1-W, T1-weighted; T2-W, T2-weighted; LGE, late gadolinium enhancement; TI, inversion time; MRA, magnetic resonance angiography; FPP, first-pass perfusion; ECV, extracellular volume*.

### Rheumatoid Arthritis

#### General Description

RA is a chronic autoimmune inflammatory arthritis that affects the joints and other systems, including the heart and CV system, even associated with the early development of atherosclerosis (102, 103) and increased vascular morbidity and mortality (104–109). CVD is the most common cause of death in the general population; these patients have up to 50% mortality and twice the risk of myocardial infarction. This is mainly related to individual genetics and a chronic and prolonged inflammatory activity where specific autoantibodies, cytokines, necrosis tumor factors, and matrix-degrading enzymes contribute to heart and atherosclerotic damage (110).

**Figure 4:**
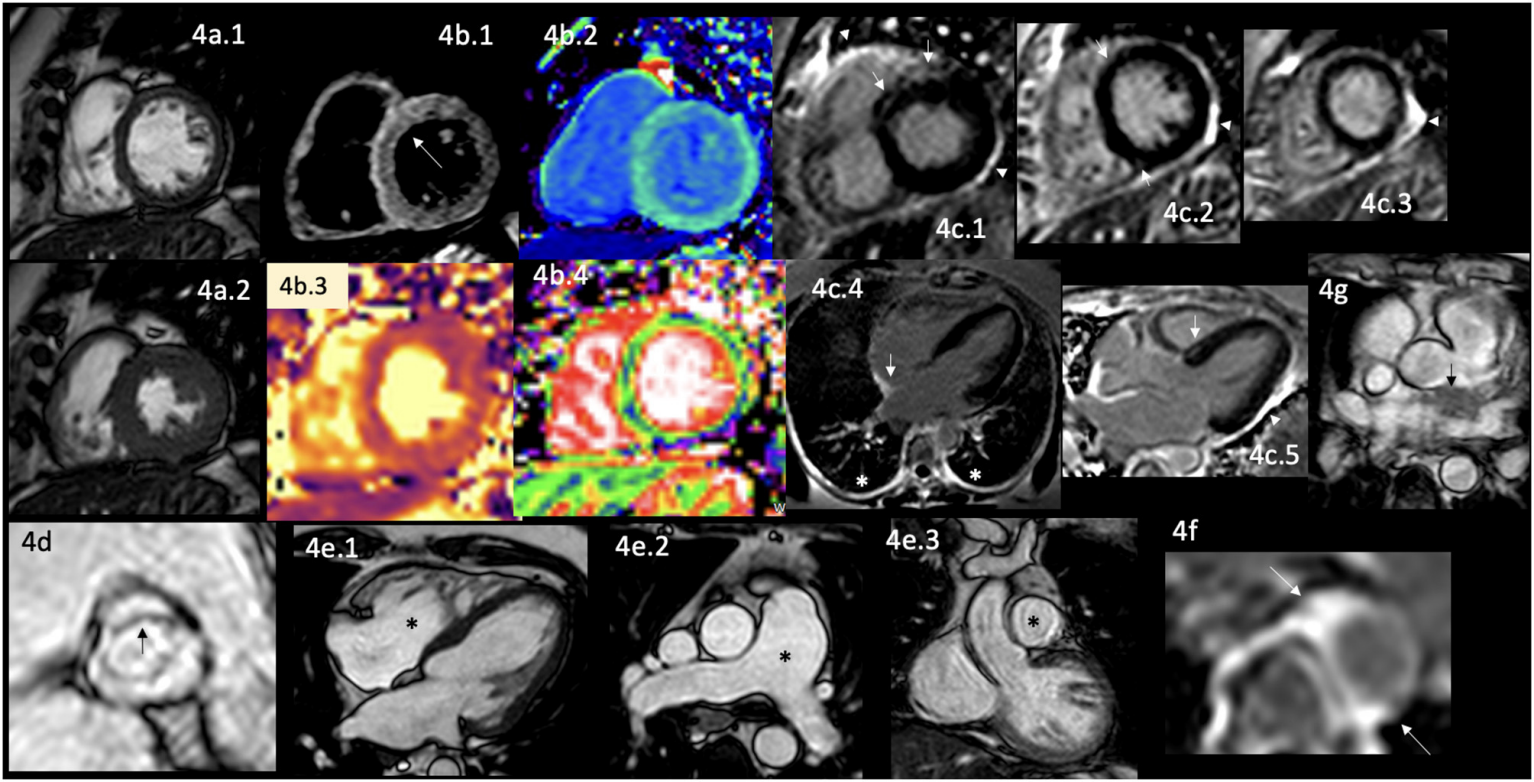
CMR findings in AR. A panel figure demonstrates varying findings from a comprehensive CMR study at a 3.0 Tesla scanner of RA with PH and arrhythmias. **(a)** SSFP sequence still cine images in short-axis view at the mid-ventricular level in end-diastole **(a.1)** and in end-systole **(a.2)** show normal global and regional left ventricular functions, mass, and wall thickness, LVEF 64% and moderately reduced systolic global RV function with global hypokinesia and mild hypertrophy (RV mass index 95 g/m^2^), RVEF 44%. **(b.1)** T2-W STIR sequence in short-axis view at a mid-ventricular level showing mildly increased signal intensity in the anterior, anteroseptal, and inferoseptal segments (arrow) with a myocardial/skeletal muscle ratio of 2.4 suggesting mild myocardial edema. **(b.2)** T1 mapping with a native T1 of 1,212 ms (normal), **(b.3)** T2 mapping with a T2 time of 42 msec (upper limit of normal) and **(b.4)** the ECV map corresponding to an elevated 52%. **(c)** LGE PSIR sequence. **(c.1–3)** Short-axis views at basal **(c.1)**, mid **(c.2)**, and apical **(c.3)** levels, and long-axis views in four-chambers **(c.4)** and three-chambers **(c.5)** projections. The arrows in these images show areas of focal fibrosis as midventricular LGE in the anteroseptal segment in **(c.1,2)** and the subepicardium of the anterior segment in **(c.1)** and subepicardial in the inferior segment in **(c.2). (c.4)** Shows LGE on the upper RA wall (arrow) and both-sided pleura (asterisks). **(c.5)** Shows mid-wall LGE on the anteroseptal segment. Pericardial LGE is shown in **(c.1–3,5)** (arrowheads), consistent with pericardial inflammation in the absence of pericardial effusion. **(d)** Zoomed still cine image in SSFP sequence of the aortic valve showing cusps thickening (arrow) with a mildly reduced valvular area (1.57 cm^2^). **(e.1–3)** SSFP sequence still cine images in long-axis 4-chambers view **(e.1)** showing RV enlargement (asterisks), **(e.2)** true axial view at great vessels level showing a mildly enlarged PA (asterisks) confirmed in the corresponding coronal view **(e.3)**. **(f)** Zoom LGE PSIR image of the descending aorta showing wall artery enhancement (arrows). **(g)** SSFP sequence still cine images in the true axial view show an isointense tissue between the aortic root and the LA, probably of a granulomatous origin (arrow). CMR, cardiovascular magnetic resonance; RA, rheumatoid arthritis; SSFP, steady-state free precession; LVEF%, left ventricular ejection fraction; RVEF%, right ventricular ejection fraction; T2-W STIR, T2-weighted short-tau inversion recovery; ECV, extracellular volume; T1-W, T1-weighted; LGE, late gadolinium enhancement; PSIR, phase-sensitive inversion recovery; PA, pulmonary artery; LA, left atrium.

Therefore, many efforts to identify patients at risk of CVD have been made through different approaches. It has been demonstrated that patients have hidden or incipient damage during the AD diagnosis (110). Patients with RA have a broad spectrum of CV damage during their natural history, including the early presentation of left ventricular (LV) dysfunction (103) without other attributable causes. Early identification of cardiac abnormalities is crucial because it parallels other risk factors of this systemic disease (111–113), and the coexistence of CVD reduces survival (106, 114–116). It is also worrisome that in subclinical patients with RA, regularly evaluated with Echo, the LV global systolic function is reported as normal with diastolic dysfunction, frequently reported adequate to the age of the patient; however, when those same patients are evaluated with CMR (Figure 4a.1–2), it shows regional wall motion abnormalities with reduced circumferential strain at mid-ventricular level with LGE of non-coronary pattern often located in the inferolateral segment at basal and mid-ventricular levels (114–116).

Animal model studies have investigated the correlation between the clinical phenotypes of RA and the inflammatory state to correlate the inflammatory activity with the biomarkers and findings in non-invasive imaging modalities (117), which have been reproduced in humans with the disease (118–121).

It is common to find the association with other comorbidities that makes it challenging to identify the specific cause of damage to the CV system, increasing its risk (122). Common comorbidities are systemic arterial hypertension, diabetes mellitus (123, 124), chronic kidney disease (125), and obstructive sleep apnea (126). These comorbidities usually do not have a proper evaluation and treatment (127). Therefore, action is needed to investigate the interaction between the inflammatory state and the presence of these diseases with cardiovascular involvement (115).

#### Clinical Manifestations of CV Involvement and Pathogenesis of Cardiac Manifestations

Cardiac involvement in RA includes VHD in 9% of cases (128). The most commonly affected is the aortic valve, by thickening of its leaflets (Figure 4d) or frequently by producing stenosis (129, 130). Ischemic heart disease in 8% of cases (104, 128, 131), myocardial damage that usually remains subclinical (132, 133), and the development of HF of different degrees until reaching its congestive form in 10% of cases (104, 130, 134). Myocardial damage involves diverse causes, which are well described, including subclinical concentric LV hypertrophy, which is considered one of the earliest CV involvement (135–140), microvascular dysfunction, CAD, myocardial ischemia, myocarditis in 6% of cases (130, 141, 142) (Figure 4b.1–4), myocardial fibrosis (142, 143) (Figure 4c.1–5), dilated cardiomyopathy (141), pericardial effusion in 21% of cases (130), pericardial inflammation (Figure 4c.1–3,5), rhythm disturbances in 22% of patients of which 20% have conduction blockades (130), pleural effusion in 9% of cases (130), pleural inflammation (Figure 4c.4) and pulmonary fibrosis in 6% of cases (130). Great vessels are also involved; it is described as abnormal LGE, aortic compliance, and its pulse wave velocity (117, 144) reflecting aortic stiffness (Figure 4f).

The incidence of congestive HF is approximately 2-fold higher than the general population (134).

The pathophysiological mechanisms are related to myocardial inflammation (Figure 4b.2,4), vascular remodeling, alterations in the extracellular matrix (Figure 4b.4), edema (Figure 4b.1,3), and myocardial injury demonstrated by circulating biomarkers, histology, and non-invasive imaging findings (145); premature atherosclerosis and the coexistence with other comorbidities as mentioned before. Interestingly, some areas of myocardial fibrosis match the cardiac conduction system in patients with intracardiac blockades (Figure 4c.4) that are not attributable to CAD or system degeneration.

RA patients are at increased risk for developing PH and right HF. The underlying mechanisms are interstitial lung disease, vasculitis, and chronic thromboembolic disease (146).

There are robust biomarkers for prognosis and therapeutics based on specific CMR findings (147). It is essential to identify early PH in these patients since findings with current non-invasive strategies are available (146). Frequently, the conventional Echo measurements do not provide all relevant information, mainly related to the RV function.

#### CMR Role

It has been proven that laboratory tests and specific inflammation imaging markers can predict the incidence of CV events (145, 148–153). Therefore a comprehensive CMR study is considered a valuable and minimally invasive diagnostic tool to assess cardiac involvement in asymptomatic and symptomatic RA patients (154); since it helps in the timely monitoring of the treatment response and early detection of the various structural and functional cardiac abnormalities (113), including the acceleration of atherosclerosis from the systemic inflammation (148, 155–158).

CMR can identify the indirect signs of PH in the same comprehensive study done to analyze the rest of the cardiac involvement of this disease (Figure 4e.1–3), constituting one of the complete non-invasive diagnostic modalities since it enables the evaluation of function and morphology of PA and RV (147). The findings are the gold standard measurements of RV function (147), systolic shift of the interventricular septum toward the LV, flow measurements, strain analysis with myocardial feature tracking, which can detect RV dysfunction even with normal RV ejection fraction (147), 4D-flow allowing more accurate and multilocation flow analysis simultaneously (159), LGE in RV which is associated with worse prognosis (147), T1 mapping that detects interstitial fibrosis even without evident LGE (147).

Interestingly, like in those patients with cardiac sarcoid, in some cases of RA, it is possible to visualize mediastinal extracardiac tissue (Figure 4g).

See Table 4 for detailed CMR offerings to RA and Table 1 for pros and cons over other imaging modalities.

**Table 4:** CMR offerings to the RA.

**Cardiac manifestation**	**CMR utility**	**Sequences**
**What has CMR to offer in rheumatoid arthritis?**		
LV dysfunction	The gold standard for LV function.	Cine—SSFP Strain, diffusion tensor
Myocarditis	Identification and quantification. Follow up free of radiation.	T2-W-STIR T1, T2-mapping, ECV LGE-PSIR
Myocardial fibrosis	Identification, location, and quantification. Follow up free of radiation. Prognostic information.	T1, T2-mapping, ECV LGE-PSIR
Heart failure	The gold standard for RV and LV function. Fibrosis pattern, location, severity, and quantification. Follow up free of radiation. Prognostic information.	Cine – SSFP Strain, diffusion tensor T1, T2 mapping, ECV LGE-PSIR
Vascular involvement	Great vessels – Aortic involvement	Cine – SSFP, cine-FGE 4D-flow, MRA

*SSFP, steady-state free precession; T2-W, T2-weighted; STIR, short tau inversion recovery; LGE, late gadolinium enhancement; PSIR, Phase-sensitive inversion recovery; MRA, magnetic resonance angiography; ECV, extracellular volume*.

### Systemic Sclerosis

#### General Description

SSc is a connective tissue disease classified based on skin involvement into two forms: diffuse cutaneous systemic sclerosis (DcSSc) and a focal, called limited cutaneous systemic sclerosis (LcSSc), previously known as CREST syndrome (160). LcSSc and DcSSc are associated with various systemic manifestations and autoantibody positivity. ANA may be present in more than 90% of cases of systemic sclerosis, and at least one of the more specific autoantibodies (anti-centromere, anti-SCL70, and anti-RNA polymerase III) is present in up to 70% of cases (161). The organs most frequently affected by scleroderma are the skin, gastrointestinal tract, lungs, kidneys, skeletal muscle, and the heart (161).

#### Clinical Manifestations of CV Involvement and Pathogenesis of Cardiac Manifestations

**Figure 5:**
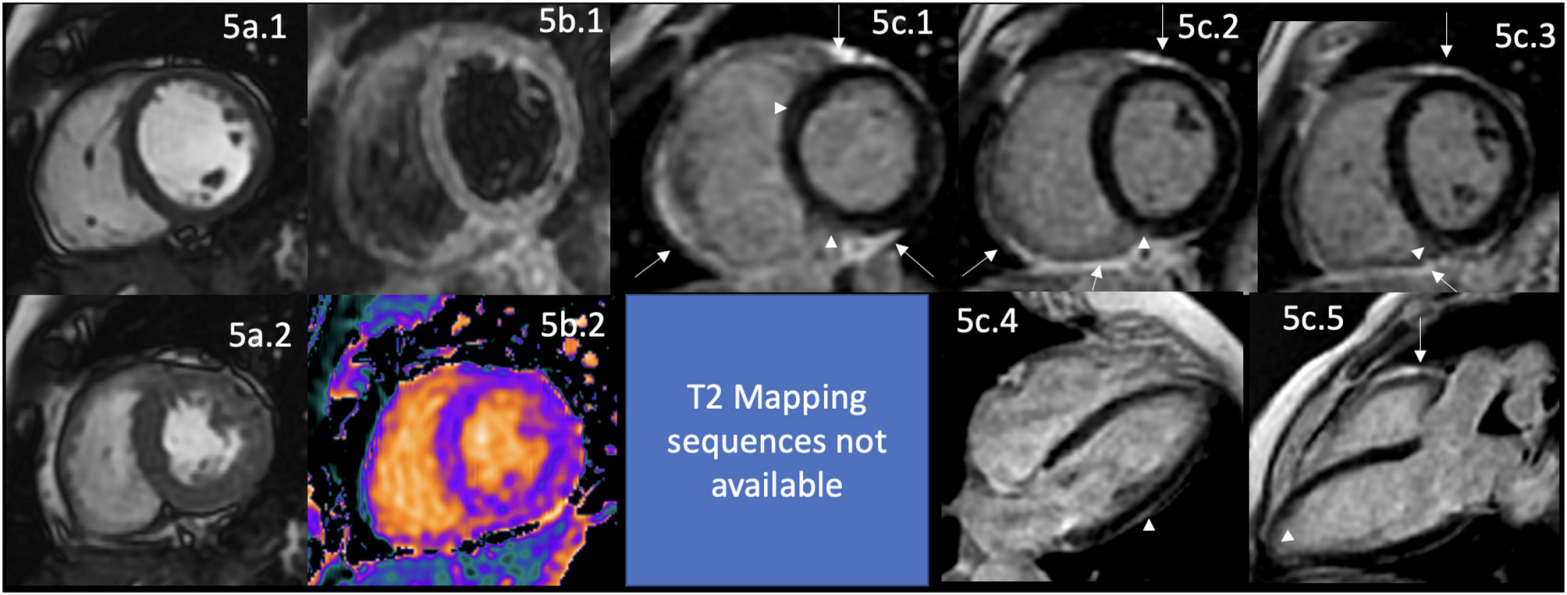
CMR findings in SSc. A panel figure demonstrates varying findings by a comprehensive CMR study at 1.5 Tesla scanner of SS. **(a)** SSFP sequence still cine images in short-axis view at the mid-ventricular level in end-diastole **(a.1)** and end-systole **(a.2)** shows normal global and regional functions of both ventricles, LVEF 57% and RVEF 56%. **(b.1)** T2-W STIR sequence in short-axis view at the mid-ventricular level of normal myocardium and myocardial/skeletal muscle ratio. **(b.2)** T1 mapping with a native T1 of 998 ms (mildly increase) and a calculated ECV of 32% (mildly increase). **(c.1–5)** LGE PSIR sequence. **(c.1–3)** Short-axis views at basal **(c.1)**, mid **(c.2)**, and apical **(c.3)** levels, and long-axis views in 4-chambers **(c.4)** and 3-chambers **(c.5)**. The arrowheads in these images show areas of focal fibrosis as mid-wall LGE of the anteroseptal segment in **(c.1)** and the subepicardium of the inferior segment in **(c.1–3)** in the basal and mid anterolateral segments in **(c.4)** and subendocardial in the LV apex in **(c.5)**. In addition, the same set of images shows LGE of the pericardium without effusion (arrow), suggestive of pericardial inflammation. CMR, cardiovascular magnetic resonance; SS, systemic sclerosis; SSFP, steady-state free precession; LVEF%, left ventricular ejection fraction; RVEF%, right ventricular ejection fraction; T2-W STIR, T2-weighted short-tau inversion recovery; ECV, extracellular volume; LGE, late gadolinium enhancement; PSIR, phase-sensitive inversion recovery.

Cardiac involvement is reported high in histopathological reports as in CMR studies, reaching 75–80% of patients with this disease (162). The cardiovascular clinical presentation most commonly includes the lungs with nonspecific interstitial pneumonitis, interstitial pneumonia, and PH (163). In the heart (Figure 1), both forms of SSc (164) includes pericardial inflammation (Figure 5c.1–5) with clinical presentation of pericarditis in 10–20% of cases (165), pericardial effusions with fibrosis (Figure 5c.1–5) and thickening that reaches constrictive pericarditis, and myocardial involvement, with edema (Figure 5b.1) presenting as myocarditis that could be even fulminant (164, 166), and fibrosis (Figure 5c.1–5), which characteristically affects both ventricles (166, 167) that progresses to conduction disturbances, such as conduction blockades, atrioventricular or intraventricular in 28–52.8% of cases on resting ECG or 38–56% of cases on 24-h Holter monitoring (168) or ventricular arrhythmias; and progressive cardiac dilatation and systolic-diastolic dysfunction ending up in HF (164). Diastolic dysfunction is the most common finding, reported in 30 (165) to 44% of cases (169), may be related to the broader use of Echo as the initial, and sometimes, a unique diagnostic tool for the evaluation of the involvement of the heart in this disease. Some reports also describe VHD (167).

The pathophysiological mechanisms affect mainly the lungs with vascular damage, autoimmunity, loss of pulmonary microvasculature (163), and tissue fibrosis. It is non-inflammatory proliferative/obliterative vasculopathy followed by interstitial/vascular fibrosis (170). Perivascular inflammatory infiltrates of CD4+ T lymphocytes may be seen early in the disease but are absent at long-standing stages. This vasculopathy is characterized by mild intimal proliferation and basement membrane thickening, loss of vascular endothelial cadherin, platelet aggregation, and microthrombus formation. The presence of vasculitis or immunocomplex deposition is infrequent. Over time in long-standing cases, there is extensive perivascular fibrosis, progressive luminal occlusion, and tissue fibrosis (171), making the vascular reactivity fixed and unreversible. Similar pathophysiology as lung vessels affects the microvasculature in the heart producing myocardial necrosis and chronic small vessel vasculopathy similar to the one in Raynaud's phenomenon (167).

It has been reported that the positivity of Anti-topoisomerase I autoantibodies is associated with cardiac involvement (172). The clinical markers of risk for the development of systolic dysfunction are the male gender, older ages, the coexistence of digital ulcers, and skeletal muscle involvement (172).

#### CMR Role

CMR evaluation of these patients allows for assessing the right-sided heart. It shows that, even in the absence of PH, there are hemodynamic abnormalities manifested as right atria and ventricle enlargement, which increases progressively along with the vascular resistance and the pulmonary artery systolic pressure (PASP) with the establishment of PH and finally with RV dysfunction in 5–10% of cases (165, 169). CMR cannot measure the PASP directly; however, some indirect findings orient to the presence of PH and correlate well with the invasive measurement of PASP; one important finding is the systolic shift of the interventricular septum toward the LV, when this finding is observed in CMR the PASP is usually higher than 67 mmHg; therefore, the patient is with at least moderate PH (173).

Interestingly, even with the detection of myocardial edema (Figure 5b.1), focal and diffuse fibrosis (Figure 5c.1–5) by CMR, and the presence of a certain degree of LV dysfunction present in 5% of cases (165), the clinical presentation of HF is uncommon in these patients, with only 5% manifesting the same (174). LGE in SSc has a typical pattern of non-ischemic cardiomyopathy (Figure 5c.1–5), with non-coronary distribution as in other ADs, with a linear mid-wall distribution, even in subclinical and early-stage cases; without significant impairment of cardiac function at that time (175) (Figure 5a.1,2). As in ischemic patients with a higher LGE burden, ventricular arrhythmias have a stronger association, supporting the hypothesis of a fibrotic substrate for this kind of rhythm disturbance. According to the SAnCtUS score, the significant T2-STIR myocardium/skeletal muscle ratio (>2.3) and the presence of more than 5% of LGE of the LV mass can predict the development of potentially life-threatening ventricular arrhythmic events (176).

T1 mapping and ECV values are significantly higher in patients with SSc than in healthy controls (Figure 5b). In addition, a higher burden of diffuse myocardial fibrosis measured by mapping techniques, higher native T1 value (Figure 5b.2), and increased ECV have been associated with more severe interstitial lung disease (177). The potential role of this technique in avoiding the additional risks and costs of using the current gold standard method, the endomyocardial biopsy (178), remains unclear. Still, well-designed studies are helping to answer that question correctly.

The UK Systemic Sclerosis Study Group proposed a comprehensive algorithm that classifies patients as asymptomatic, at-risk, and symptomatic for cardiac complications, including the standard assessment including patient history, blood pressure, lipid profile, and HbA1c, ECG, Echo, a core set of parameters to be measured at each Echo and laboratory parameters; the follow-up intervals are determined by the risk categorization. In this context, CMR is recommended in a more individualized analysis, depending on the clinical manifestation of each patient independent of their risk categorization in the algorithm (179). See Table 5 for detailed CMR offerings to SSc and Table 1 for pros and cons over other imaging modalities.

**Table 5:** CMR offerings to the SSc.

**Cardiac manifestation**	**CMR utility**	**Sequences**
**What has CMR to offer in systemic sclerosis?**		
Myocarditis	Identification and quantification. Follow up free of radiation.	T2-W-STIR T1, T2-mapping, ECV LGE-PSIR
Pericarditis	Identification. Measurement of pericardial thickness. Detection of inflammation. Detection of constriction. Follow up free of radiation.	Cine—SSFP Free-breathing real-time cine T1-W, T2-W STIR LGE-PSIR
Pulmonary hypertension	The severity and hemodynamic significance. Orientation to the potential etiology.	Cine—SSFP Real-time 4D-flow MRA
RV dysfunction	The gold standard for RV function	Cine—SSFP Strain
LV dysfunction	Diastolic	PhC T1, T2 mapping, ECV LGE-PSIR
	Systolic: The gold standard for LV function.	Cine—SSFP Strain, diffusion tensor

*SSFP, steady-state free precession; T2-W, T2-weighted; STIR, short tau inversion recovery; LGE, late gadolinium enhancement; PSIR, Phase-sensitive inversion recovery; MRA, magnetic resonance angiography; ECV, extracellular volume; PhC, phase contrast*.

### Adult-Onset Still'S Disease

#### General Description

AOSD is a non-hereditary, non-organ specific, polygenic autoinflammatory disease that affects young adults in a bimodal distribution with an average age of 15–25 and 36–46 years old and an incidence of 0.16–0.4/100,000 individuals (180). Its pathogenic mechanism involves a genetic background with triggering factors that generate a dysregulated immune activation with overproduction of inflammatory cytokines, principally IL-1, IL-6, and IL-18, and a deficiency of anti-inflammatory mechanisms (181).

Based on the Yamaguchi diagnostic criteria, the main clinical manifestations are the presence of febrile peaks, non-pruritic erythema, arthritis, polyserositis, odynophagia, lymphadenopathy, and hepatosplenomegaly. Associated with elevated serum biomarkers such as leukocytes, globular sedimentation rate, and ferritin (165).

#### Clinical Manifestations of CV Involvement and Pathogenesis of Cardiac Manifestations

It affects the CV system within the spectrum of systemic alterations (Figure 1). This disease mainly affects serosal layers; therefore, it presents with pericarditis in 37% of cases with CV involvement. Pericarditis can evolve until tamponade, but it is rare. Other described forms of cardiac involvement are endocarditis and myocarditis, which are present in 7% of cases (180, 182, 183).

Common clinical CV manifestations are chest pain, dyspnea, tachycardia, atrial gallop, pericardial rub, and pulmonary congestion; physical examination data, all manifestations according to LV systolic dysfunction and the progression to HF even to cardiogenic shock. The development of cardiogenic shock is considered the second cause of mortality in these patients, accounting for 4.2% in the acute event and up to 21% in 1 year (180, 184). Interestingly, this disease shows non-specific ST segment and T wave alterations often in the acute event (79% of cases) (185), probably related to concomitant non-obstructive involvement of coronary arteries not well described till today (180, 184, 186). Usually, myocarditis occurs early in the evolution of SD, and it has been reported in the adult-onset of SD in 54% of the cases (185).

It is not surprising that the presence of myocarditis, which is often symptomatic in 96% of cases, with electrocardiogram abnormalities mentioned above and LV dysfunction with reduced LVEF <50% in 67% of cases (185); can manifest with arrhythmias and ventricular dysfunction as it has been described, based on the cytokine storm that is part of this disease (180, 184, 186). CMR has good diagnostic accuracy in this disease (182, 187, 188) and can demonstrate the presence of myocarditis found with the endomyocardial biopsy (185). It is crucial to identify the presence and severity of cardiac involvement, mainly in myocarditis cases. Still, the LV dysfunction is not evident since the conventional therapy with steroids alone is effective only in 50% of cases with myocarditis. Few of them can evolve to a potentially life-threatening cardiogenic shock (185) which is preventable with timely detection, for example, with CMR, since the only known predisposing factors are male gender and younger age of adult-onset SD is not a rule. Myocarditis is found in this subset of AOSD. In that case, it is mandatory to adjust the medical treatment to intravenous immunoglobulins, methotrexate, anakinra, an anti-IL1 (186), and tumor necrosis factor-α (TNF- α)-blockers, which have often been effectual (185, 186).

The presence of pericarditis is reported more often and usually correlates with white blood cell count, polymorphonuclear cell count, and higher serum ferritin levels (185).

The pathogenic mechanism was hypothesized that cardiac involvement is secondary to excessive immune activation and, therefore, to the previously mentioned cytokine storm, which contributes to a partial response to the standard medical treatment (180, 184).

#### CMR Role

As mentioned above, in the early stages of the disease, the myocardial and serosal involvement could course asymptomatic, leading to a misdiagnosed entity. Despite high-level serum inflammatory biomarkers or unspecific ECG changes, it is only suspected when there is clinical evidence of HF. Therefore, the evaluation of CMR with T1-weighted, T2-weighted, and post-gadolinium enhancement allows in a non-invasive manner with good sensitivity and specificity the early detection of cardiac involvement, tissue characterization, and left ventricle function, helping in the prompt initiation of steroids, biotherapies or neurohumoral blockage in cases of systolic dysfunction since it increases morbidity and could be potentially fatal.

See Table 6 for detailed CMR offerings to SD and Table 1 for pros and cons over other imaging modalities.

**Table 6:** CMR offerings to the AOSD.

**Cardiac manifestation**	**CMR utility**	**Sequences**
**What has CMR to offer in adult-onset Still's disease?**		
LV dysfunction	The gold standard for LV function	Cine—SSFP Strain, diffusion tensor
Myocarditis	Identification and quantification. Follow up free of radiation.	T2-W-STIR T1, T2-mapping, ECV LGE-PSIR
Pericarditis	Identification. Measurement of pericardial thickness. Detection of inflammation. Detection of constriction. Follow up free of radiation.	Cine—SSFP Free-breathing real-time cine T1-W, T2-W STIR LGE-PSIR
Tamponade	Identification Assessment of severity and hemodynamic significance, detection of inflammation.	Cine—SSFP or SSh Free-breathing real-time cine LGE-PSIR

*SSFP, steady-state free precession; T2-W, T2-weighted; STIR, short tau inversion recovery; LGE, late gadolinium enhancement; PSIR, Phase-sensitive inversion recovery; ECV, extracellular volume; SSh, single-shot*.

### Polymyositis and Dermatomyositis

#### General Description

An idiopathic inflammatory myopathy presents proximal, and symmetric muscle weakness due to inflammatory infiltrates in the skeletal muscle. In some cases, it has extraskeletal and muscular involvement in the skin, the lung, the joints, the heart, and distal arteries with Raynaud's phenomenon (189).

It presents after the second decade of life and is more common between 45 and 60 years; females are mostly affected with a ratio of 2:1 (190).

Genetically is associated with the major histocompatibility complex (MHC) at chromosome 6, at the HLA-DRB1^*^03:01 and HLA-B^*^08:01 alleles of the ancestral haplotype 8.1 (8.1AH) (190).

The usual clinical presentation is symmetric proximal muscle weakness (in >90% of cases in PM and >50% in DM) with skin manifestations in DM. The onset of symptoms can be in days or more insidious, weeks or even months, with fever, malaise, weight loss, asthenia, and adynamic. Muscle atrophy is a late complication in advanced disease. In DM, the rash often precedes or accompanies muscle weakness. Cutaneous manifestations can be pathognomonic as (1) Gottron's papules, which involve extensor regions such as the knuckles, elbows, or knees, (2) heliotrope rash involving the periorbital region with or without edema, (3) erythematous rash on the chest, back in regions exposed to the sun, (4) facial erythema or a malar rash that does not spare the nasolabial fold, (5) periungual hyperkeratosis and telangiectasias, which represent rings of capillary dilation in the nail matrix, (6) dermal ulcers related to vasculopathy, (7) calcinosis of the subcutaneous tissue in the muscular fasciae, (8) lipodystrophy and hyperkeratosis with cracks in the palms (190, 191).

#### Clinical Manifestations of CV Involvement and Pathogenesis of Cardiac Manifestations

Cardiac involvement in Polymyositis (PM) and dermatomyositis (DM) derives from exposure to an inflammatory process for a specific time, which causes structural and functional alterations of the heart. The affected cardiac structures are the valves, the conduction system, the myocardium, the endocardium, the pericardium, and the pulmonary and coronary arteries (192) (Figure 1).

Heart valves are affected due to the ongoing inflammatory process, causing leaflet thickening and dysfunction. Although rare, the aortic (46.7%) and the mitral (20%) valves are most frequently involved, often producing stenosis. Interestingly, mitral stenosis could be the primary manifestation in up to 6.7% of cases depicted by Echo studies (193).

The histologic substrate is lymphocytic infiltration of the conduction system and the fibrosis of the sinoatrial node (194).

Aside from the VHD previously described, it is common to identify conduction system disturbances at different levels, manifested with a diverse degree of severity and location of His bundle and its branches in 33–72% of cases (194) or arrhythmias identified in 52–88% of cases, where the most common are supraventricular tachycardia, premature ventricular complexes, ventricular tachycardias, and atrial fibrillation. Frequently, supraventricular tachycardias, including atrial fibrillation, are present in 12–50% of patients developing myocarditis (195).

In PM, it is frequent to encounter the presence of LV hypertrophy easily assessed by Echo (194). Interestingly, many patients with this disease and cardiac involvement remain subclinical. Therefore, high suspicion is necessary, and the role of CMR is crucial for the timely identification of cases likely to develop these complications so they can receive appropriate, timely, and specific treatment responsive to appropriate immunosuppressive therapy (196, 197).

In PM and DM, it is common to identify HF in a variable range from 32 to 77%, which could be diastolic or even severe systolic dysfunction (197). Patients have LV dysfunction.

Myocarditis has been described in up to 8% of cases (196). Myocardial ischemia has been described in 26% of patients with PM/DM; the main symptoms are angina (4%) and exertion dyspnea (18%), although the latter could be associated with ventricular dysfunction (198). The underlying mechanisms are related to the inflammatory state that produces acceleration in coronary atherosclerosis, rapid progression of CAD, and making early stages of atheromatous plaques prone to instability favoring the presence of an acute coronary syndrome due to their rupture (198). LV dysfunction is associated with an increase in ventricular filling pressures contributing to a reduction in coronary perfusion pressure, predisposing patients to developing myocardial ischemia independent of the presence of CAD (198).

As in other ADs, the association between PM/DM and aseptic endocarditis has been described. However, in this case, it is an infrequent entity with possibly catastrophic consequences if not diagnosed in time (199).

Pericarditis is reported in 4–25% of adults with PM/DM, and most of them remain asymptomatic and hemodynamically insignificant (200). The exception is in those patients with the PM that are positive for anti-Jo, anti-Mi, and anti-SRP antibodies (201). The anti-Jo-1 is the primary antibody that defines the antisynthetase syndrome (202, 203), and the other antibodies support the diagnosis. The most specific antibody for DM is the anti-Mi-2 (204), where the anti-SRP is present in the immune-mediated necrotizing myopathy variant (205). In the antisynthetase syndrome, the incidence of pericarditis is higher, 50%, and it tends to have more significant hemodynamic repercussions, even reaching cardiac tamponade (195).

PH is a life-threatening entity that culminates in severe right ventricular dysfunction and HF. In PM patients with the antisynthetase syndrome, PH is present in 8% of cases, which are very ill (195).

#### CMR Role

CMR shows LGE in the non-coronary territory consistent with non-ischemic cardiomyopathy, reported in 62.3% of cases consistent with myocarditis (197). CMR findings are compatible with myocardial inflammation, mainly located in the inferior and lateral segments of the LV (197).

The presence of LGE in PM/DM has similar behavior to Duchenne muscular dystrophy (206, 207). It needs careful interpretation since it appears that the presence of LGE could have a protective effect on LV systolic global function. In a large cohort of PM/DM, the authors found reduced LV function in 17% of the patients, and all showed LGE; however, in the remaining patients, with normal LV systolic global function, up to 54.5% of them had LGE. This suggests that fibrosis may be an earlier change in the myocardium and precedes the establishment of LV systolic global dysfunction (197). See Table 7 for detailed CMR offerings to PM/DM and Table 1 for pros and cons over other imaging modalities.

**Table 7:** CMR offerings to the PM/DM.

**Cardiac manifestation**	**CMR utility**	**Sequences**
**What has CMR to offer in polymyositis/dermatomyositis?**		
Valvular heart disease	Assessment of hemodynamic significance. Reproducible follow-up.	Cine—SSFP, cine-FGE PhC, 4D-flow
Pulmonic hypertension	The severity and hemodynamic significance. Orientation to the potential etiology.	Cine—SSFP Real-time cine 4D-flow MRA
Myocardial edema	Identification and quantification Follow up	T2-W, T2-W-STIR T2-mapping
Myocarditis	Identification and quantification. Follow up free of radiation.	T2-W-STIR T1, T2-mapping, ECV LGE-PSIR
Myocardial inflammation	Identification and quantification. Follow up free of radiation.	T1-W pre, and post-Gd T1 mapping, ECV LGE-PSIR
RV dysfunction	The gold standard for RV function	Cine—SSFP Strain
LV dysfunction	The gold standard for LV function	Cine—SSFP Strain, diffusion tensor

*SSFP, steady-state free precession; FGE, fast gradient echo; T2-W, T2-weighted; STIR, short tau inversion recovery; LGE, late gadolinium enhancement; PSIR, Phase-sensitive inversion recovery; ECV, extracellular volume; MRA, magnetic resonance angiography; Gd, gadolinium*.

### Eosinophilic Granulomatosis With Polyangiitis (Formerly Churg-Strauss Syndrome)

#### General Description

Churg-Strauss syndrome, renamed eosinophilic granulomatosis with polyangiitis (EGPA), is a systemic vasculitis characterized by disseminated necrotizing vasculitis and extravascular granulomas (34). EGPA is a rare vasculitis, often insidious and underestimated, classified as small and medium-sized vessel vasculitis associated with antineutrophil cytoplasmic antibodies (ANCA) and the hypereosinophilic syndrome (208). It affects multiple organ systems, especially the lungs. Its pathophysiology includes the presence of hypereosinophilia, inflammation of blood vessels producing vasculitis, and nodular inflammatory lesions as granulomas.

#### Clinical Manifestations of CV Involvement and Pathogenesis of Cardiac Manifestations

Heart involvement has been described in 15–60% of cases, mainly in those antineutrophil cytoplasmic antibodies (ANCA) negative (208) and includes myocarditis, pericarditis, conduction disturbances manifested by different types of arrhythmias (most commonly heart block), valvular heart disease, intracavitary thrombosis, coronary arteritis, LV dysfunction, and HF (208–210) (Figure 1). The timing for CV involvement varies; it is usually an early manifestation of the disease but could be a later presentation (208). An important problem is that almost 40% with cardiac involvement remain asymptomatic and have no significant ECG abnormalities (208, 210). Patients with cardiac involvement have a poor prognosis and are responsible for 50% of the deaths of this disease (208–212). Therefore, all asthmatic patients with dyspnea, vasculitis, and hypereosinophilia suspected of EGPA cardiac involvement should be considered (207) since early identification and diagnosis of the cardiac involvement and proper treatment may prevent the progression of the cardiac disease (203).

The early diagnosis of cardiac involvement is crucial because the prognosis is poor once HF is established (213), even though some are still reversible (210, 212).

Myocarditis in this syndrome is the most severe manifestation (210, 212). It could be the first manifestation and eventually cause a fatal outcome (212, 214), presenting early or late in the disease in 59% of cases (210, 212, 215). It is typically characterized by myocardial infiltration predominantly of eosinophils, mainly caused by eosinophilic granule proteins, specifically the eosinophil cationic protein (216). It has features that should raise suspicions, such as reduced global systolic function with diffuse LV hypokinesia, increased wall thickness, total LV mass with marked myocardial edema, and diffuse subendocardial eosinophilic infiltrate (209, 216). Myocardial injury directly affects eosinophil-mediated necrosis and induction of apoptosis rather than myocardial vasculitis (211).

Its response to treatment is variable (207, 209, 210) and sometimes results in cardiogenic shock (217). A systematic review of the literature showed that myocarditis is more prevalent at younger ages. The worst prognosis is at younger ages; males are affected at younger ages than females. Usually, the patients have a previous history of severe asthma; when the eosinophils are >20% of the white blood cells count, they start to infiltrate into the myocardium and are associated with negative ANCA status (206, 213, 216).

Intracavitary thrombosis is attributed to the subendocardial eosinophilic infiltration in localized ventricular segments with impaired function (212) and its procoagulant effect in the hypereosinophilic state. Thrombosis could be localized or massive, involving both ventricles (209, 211).

The involvement of the pericardium could be as inflammation with pericarditis or, more often, as the pericardial effusion of varying degrees of severity reported in 41% of the cases with cardiac involvement (210, 212). Pericardial involvement could be isolated, have a more benign presentation, might cause congestive symptoms, rarely tamponade (215, 218), and even atraumatic intrapericardial thrombosis (219).

The conduction disturbances are frequent with cardiac involvement; however, life-threatening arrhythmias are rare and often asymptomatic (220). Complete heart block has rarely been described (221, 222). The pathophysiological mechanism behind ventricular tachycardias in EGPA is abnormal automaticity, possibly due to myocardial ischemia secondary to necrotizing vasculitis of small and medium-size arteries (220) and heart block due to infiltration of the myocardium and the His-Purkinje system (221). These arrhythmias are one of the leading causes of sudden cardiac death in these patients (220).

VHD is presented in as high as 73% of cases (212); however, it mostly only affects the aortic and mitral valves to a mild degree, but some severe cases have been described, usually mitral regurgitation (221, 223). The pathophysiology of the VHD shows necrotizing granulomatous inflammation with eosinophils infiltrating the valve leaflets, with marked thickening, in part due to fibrosis and mainly to an intense inflammatory reaction for the aortic valve (221) and the mitral valve, also related to endomyocardial fibrosis involving the papillary muscles (221, 224).

LV dysfunction has been described in as high as 50% of the cases (210, 212). Patients that develop myocarditis have less function recovery and a worse prognosis (210, 212). Studies with Echo and CMR showed that in addition to the systolic dysfunction, they have impairment of ventricular relaxation (223). The pathophysiology of that dysfunction has been suggested as myocardial edema, fibrosis (218), and eosinophilic myocardial infiltration (225); but can coexist with epicardial coronary vasculitis and resultant MI (218, 226).

RV dysfunction accompanying LV dysfunction is prominent in EGPA, as demonstrated by Echo (223) and CMR. Still, the solo presentation is highly unusual, with the typical 3-layer appearance and thrombus formation with the subendocardial LGE (227).

HF is uncommon (228–230) in around 4% of cases with EGPA (212). Still, some patients develop dilated cardiomyopathy, which may be reversible with early diagnosis and proper treatment, or could die due to HF. Interestingly, these patients are ANCA negative and have high eosinophil counts (212). These patients have intracardiac thrombus, which can embolize the brain and be the origin of stroke, an independent cause of associated brain vasculitis (230).

Coronary artery involvement in EGPA is often with arteritis (209) and rarely vasospasm (231–234). The pathophysiological mechanism is myocardial ischemia due to the sudden severe reversible vasoconstriction of a normal or diseased epicardial coronary artery (231, 235). Vasospasm has been suggested due to eosinophilic infiltration of the coronary artery wall that directly stimulates vascular smooth muscle contraction and adventitial nerve fibers (236) by their proteins and vasoactive cytokines (231, 237). Other coronary artery abnormalities are due to direct damage to the vascular tissues that predisposes it to dilatation, aneurysmal formation (233) with a higher propensity to dissection (238), and thrombus formation (239), which finally produces fibrosis of the intima and media layers of the vessel wall (236). These patients have a higher risk of recurrent coronary events despite the medical treatment for conventional atherosclerotic coronary vasospasm (237).

Pulmonic venous thrombosis has been reported with a prevalence of 8.1% (240) that could present at first diagnosis or remission (241). It presents in ANCA negative patients (242). The pathophysiological mechanisms remain unclear and speculative (243), but it seems to be related to the prothrombotic properties of the eosinophil granule proteins, such as the cationic proteins that can bind the Hageman Factor (XII) and activate the intrinsic pathway of coagulation and interfere with the anticoagulant activity of endogenous heparan sulfate (*in vitro*) (244), along with the potential role of other eosinophil products such as the hypothiocyanous acid by the induction of tissue factor activity (245).

#### CMR Role

CMR allows functional analysis along with the pathophysiological assessment of the different components of the CV involvement described in detail in the above section by the detecting myocardial edema in the myocarditis, identifying and quantifying myocardial fibrosis, and the scars related to arteritis or coronary arteries occlusions (246).

LGE in EGPA can be at mid-wall, epicardial, transmural, and often subendocardial locations (247, 248). These former two mainly with coronary artery distribution secondary to the arteritis, spasm, or even thrombotic occlusions (246). Using edema sequences, it is possible to discriminate the “age” of the LGE in that an acute lesion will have matching areas of myocardial edema and LGE, while chronic lesions will only show LGE without the corresponding matching edema area. Currently, the edema sequence using a T2-W image can be improved in detecting edema by using quantitative T2 mapping (249). Related to the diffuse fibrosis presence in this disease, it is helpful to include T1 mapping sequences and the ECV that has been demonstrated to have a good correlation with interstitial myocardial fibrosis in histology and clinically with the decrease of LVEF% (250) even before other methods such as Echo, and functional CMR would detect. See Table 8 for detailed CMR offerings to EGPA and Table 1 for pros and cons over other imaging modalities.

**Table 8:** CMR offerings to the EGPA.

**Pathophysiology**	**CMR offer**	**Sequences**
**What has CMR to offer in eosinophilic granulomatosis with polyangiitis?**		
Myocarditis	Identification and quantification. Follow up free of radiation.	T2-W-STIR T1, T2-mapping, ECV LGE-PSIR
Pericarditis	Identification. Measurement of pericardial thickness. Detection of inflammation. Detection of constriction. Follow up free of radiation.	Cine—SSFP Free-breathing real-time cine T1-W, T2-W STIR LGE-PSIR
Arrhythmias	Substrate identification, location, and quantification. Prognostic information. Follow up free of radiation.	T1, T2-mapping, ECV LGE-PSIR
Valvular heart disease	Assessment of hemodynamic significance. Reproducible follow-up.	Cine—SSFP, cine-FGE PhC, 4D-flow
Intracoronary thrombosis	Coronary arteries—atherosclerosis, thrombosis, and vasospasm.	Cine SSFP, Stress FPP LGE-PSIR, MR coronary angiography
	Microvascular dysfunction	Stress FPP LGE-PSIR
Coronary arteritis	Inflammation and thrombosis.	Cine SSFP, Stress FPP LGE-PSIR, MR coronary angiography. T1-W, T2-W, T2-STIR.
	Microvascular dysfunction	Stress FPP LGE-PSIR
LV dysfunction	The gold standard for LV function	Cine—SSFP Strain, diffusion tensor
RV dysfunction	The gold standard for RV function	Cine—SSFP Strain
Heart failure	Systolic: The gold standard for RV and LV function. Fibrosis pattern, location, severity, and quantification. Follow up free of radiation. Prognostic information.	Cine—SSFP Strain, diffusion tensor T1, T2 mapping, ECV LGE-PSIR
	Diastolic	PhC T1, T2 mapping, ECV LGE-PSIR
Coronary vasospasm	Vasospasm and myocardial ischemia.	Cine SSFP, Stress FPP LGE-PSIR, MR coronary angiography. Stress FPP LGE-PSIR
Peripheral venous thrombosis	Thrombus identification in different organs and vascular territories in the same study.^*^	T1-W, T2-W, PD, T2-STIR, SSFP, FGE, TOF, PhC, 4D-flow.

* *Except for extremities and the brain since those require a dedicated venous MR study of each location*.

*SSFP, steady-state free precession; T1-W, T1-weighted; T2-W, T2-weighted; ECV, extracellular volume; T2W-STIR, T2 weighted short-tau inversion recovery; FPP, first-pass perfusion; FGE, fast gradient echo; LGE, late gadolinium enhancement; PSIR, phase-sensitive inversion recovery; MR, magnetic resonance; PD, proton density; TOF, time of flight*.

### Dress Syndrome

#### General Description

DS is defined as Drug Reaction with Eosinophilia and Systemic Symptoms (251). DS is a severe, idiosyncratic reaction to a drug characterized by a prolonged latency period. There are a variety of non-cardiac manifestations such as fever, rash, lymphadenopathy, eosinophilia, and other systemic presentations. Cardiac involvement is reported rarely; however, its actual incidence remains unknown because it is frequently misdiagnosed (252) (Figure 1). DS has been associated with some autoimmune substrates, mainly with inflammatory polyarthritis, seronegative spondyloarthritis (253), and ankylosing spondylitis; but also with persons prone to ADs like type 1 diabetes mellitus, Hashimoto's thyroiditis (254), autoimmune hemolytic anemia (255), autoimmune enteropathy, alopecia areata, SLE, scleroderma lesions, and rheumatoid arthritis in the future (256).

#### Clinical Manifestations of CV Involvement and Pathogenesis of Cardiac Manifestations

**Figure 6:**
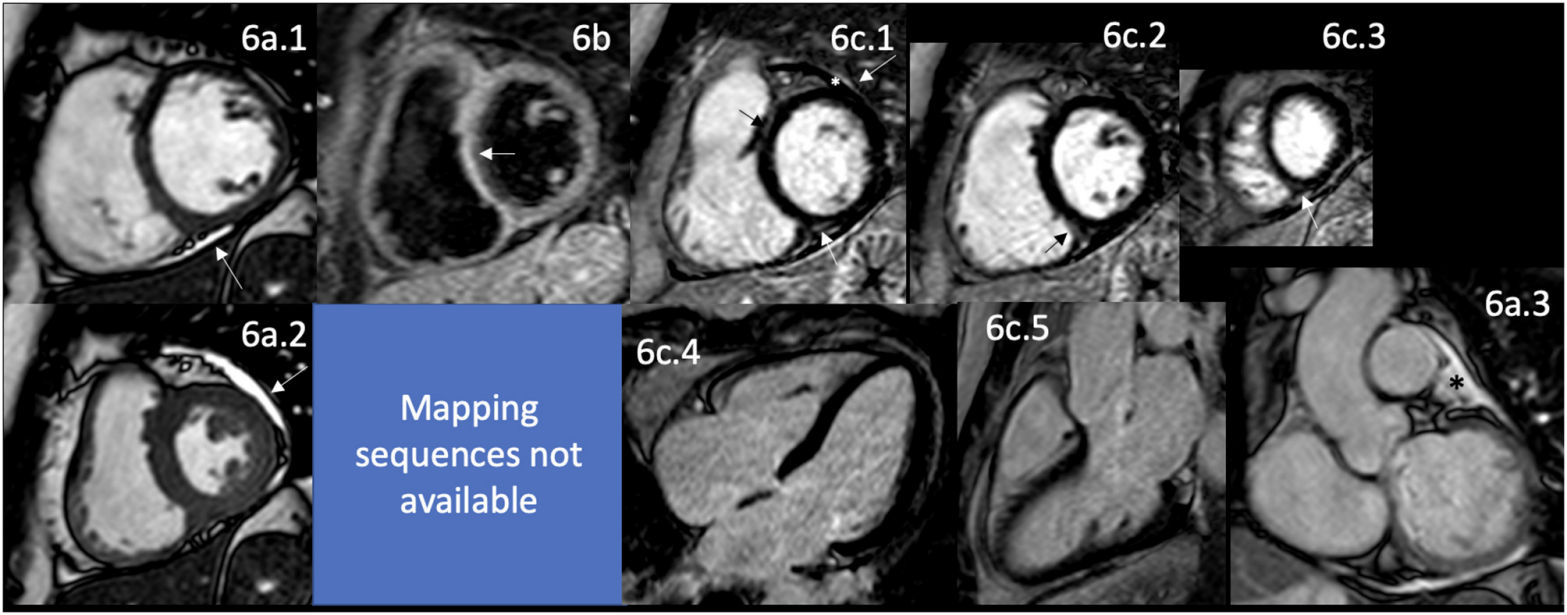
CMR findings in DS. A panel figure demonstrates varying findings from a comprehensive CMR study at a 1.5 Tesla scanner of DS. **(a)** SSFP sequence still cine images in short-axis view at the mid-ventricular level in end-diastole **(a.1)** and end-systole **(a.2)** show normal global and regional functions of the left ventricle, LVEF 63% and mildly reduce systolic global RV function and mild global hypokinesia RVEF 45%. **(a.3)** In a coronal view showing the mild pericardial effusion surrounding the heart (asterisks). **(b)** T2-W STIR sequence in short-axis view at a mid-ventricular level showing increased signal intensity in the septal segments (arrow) with a myocardial/skeletal muscle ratio of 2.3, suggesting mild myocardial edema. **(c.1–5)** LGE PSIR sequence. **(c.1–3)** Short axis views at basal **(c.1)**, mid **(c.2)**, and apical **(c.3)** levels, and long-axis views in four-chambers (**c.4**) and three-chambers (**c.5**). The arrows in these images show areas of focal fibrosis as midventricular LGE of the anteroseptal and inferior segments in **(c.1)** and in the subepicardium of the inferior segment in **(c.2,3). (c.1)** Shows mild pericardial effusion (asterisks) and trivial pericardial enhancement (arrow), suggesting minimal pericardial inflammation. CMR, cardiovascular magnetic resonance; DS, DRESS syndrome; SSFP, steady-state free precession; LVEF%, left ventricular ejection fraction; RVEF%, right ventricular ejection fraction; T2-W STIR, T2-weighted short-tau inversion recovery; LGE, late gadolinium enhancement; PSIR, phase-sensitive inversion recovery.

Cardiac involvement varies from 4 to 21% (257) and includes myocarditis and pericarditis (258). Myocarditis can be fatal and under-diagnosed, and it may occur long after the onset of symptoms of the DS (Figure 6b, c.1–3). Its pathophysiological abnormality is myocardial edema (Figure 6b) and inflammation through activated T cell reactions resulting in cytotoxicity and eosinophil activation and recruitment of leukocytes with eosinophils (90%) and/or mononuclear cells (40%) (257, 259). DS produces hypersensitivity myocarditis and acute eosinophilic myocarditis, leading to acute necrotizing eosinophilic myocarditis, cardiac thrombosis, and fibrotic stage (Figure 6c.1–3), corresponding to transient, persistent, or even fatal LV dysfunction (257). Pericarditis (260, 261) could present isolated in rare cases or more frequently associated with myocarditis (Figure 6a.1–3,c.1); these findings are markers of severity of the syndrome.

#### CMR Role

CMR has only being proposed as part of the diagnostic algorithm as in the EGPA (262) since this syndrome is rare, and maybe underdiagnosed, published information is scarce. A case report from 2018 shows the presence of myocardial edema in an edema CMR sequence, with abnormal T2 mapping consisting with edema and patchy LGE suggesting the presence of myocardial edema, inflammation and fibrosis confirmed by histology obtained with endocardial biopsy (263). Echo can identify the LV dysfunction but not all this tissue abnormalities key to guide treatment and to stablish prognosis. See Table 9 for detailed CMR offerings to DS and Table 1 for pros and cons over other imaging modalities.

**Table 9:** CMR offerings to the DS.

**Pathophysiology**	**CMR offer**	**Sequences**
**What has CMR to offer in DRESS syndrome?**		
LV dysfunction	The gold standard for LV function	Cine—SSFP Strain, diffusion tensor
Myocarditis	Identification and quantification. Follow up free of radiation.	T2-W-STIR T1, T2-mapping, ECV LGE-PSIR
Pericarditis	Identification. Measurement of pericardial thickness. Detection of inflammation. Detection of constriction. Follow up free of radiation.	Cine—SSFP Free-breathing real-time cine T1-W, T2-W STIR LGE-PSIR

*SSFP, steady-state free precession; T2-W, T2-weighted; ECV, extracellular volume; T2W-STIR, T2 weighted short-tau inversion recovery; LGE, late gadolinium enhancement; PSIR, phase-sensitive inversion recovery*.

## Discussion

Cardiovascular involvement is increasingly recognized and documented as an essential prognosticator for managing autoimmune and inflammatory rheumatic conditions; being recognized that cardiac involvement, a well-documented poor prognostic factor, is a leading cause of mortality. However, given that the condition remains clinically silent during the early stages of the disease process and often remains undetected by routine ECG, Echo, CT, and nuclear studies, increased awareness and a better understanding of the underlying multi-system pathophysiology mechanisms are imperative, with the incorporation of a robust diagnostic non-invasive modality to ensure screening, early detection, diagnosis, prognostication, guide management and follow-up of early cardiovascular involvement in patients with ADs.

In a recent publication from Sacilotto et al. (264), they found in a small cohort that the EULAR criteria are effective at identifying patients with high CV risk and that the Doppler ultrasound in carotids and femoral arteries are tools that can be used in clinical practice to detect CV disease even in their asymptomatic stage. In addition, Aceituno-Melgar et al. (265) found Echo variables that were statistically significant in RA patients and served as early diagnosis of subclinical CV involvement, supporting that even those patients in low risk or even asymptomatic according to EULAR criteria would benefit from a deeper investigation of such involvement.

PH is a common feature of almost all AD, at least in advanced stages; however, the early signs of the pulmonic vasculature involvement are often missed until the late stages, resulting in higher morbidity and mortality rates. In PH, the RV is a critical determinant of the clinical course and response to treatment of these kinds of patients. Early detection of RV dysfunction may offer a better response to therapeutic measurements. CMR allows analysis of function, morphology, and the interactive hemodynamics between PA and RV. Most cine-CMR-derived parameters (volumes and function) and flow-CMR measurements have been suggested as powerful markers for response to treatment and prognosis (147). Recent technological advances have included clinical protocols for PH evaluation that include right ventricular mechanics such as Strain analysis with myocardial feature tracking that allows early detection of subclinical early RV dysfunction. 4D-flow techniques enhance the assessment of this PA–RV binomial allowing the patient risk stratification (266) and important prognostic information gathered for over a decade in other causes of PH (267). Contrast techniques help identify the anatomy in a conventional magnetic resonance angiography, and the same study uses the information derived from LGE imaging. The extracellular volume maps to detect, locate and quantify the myocardial fibrosis even if it is still not visible to the eyeball. The detection of fibrosis in PH is associated with poor prognosis (147, 267).

A recent meta-analysis of PH confirmed CMR as a powerful prognostic marker in PH, confirming that RV function and RV and LV volumes predict mortality and that RV volumes and function predict clinical worsening and poor response to medical treatment. Therefore, significant scientific evidence strongly supports the proposal of considering CMR as part of the diagnostic algorithm in AD (267).

CMR promises the accuracy, precision, and reproducibility required in this group of patients because of its non-invasive, non-radiation linked assessment of myocardial tissue characterization by conventional and novel multiparametric methods, the gold standard in biventricular function and volumes assessment, and ability to assess microvascular dysfunction. CMR is also excellent in evaluating valves, thrombosis, and conduction-linked myocardial disorders, besides pericardial, vascular, and aortic involvements linked to ADs.

Recent advances in CMR, including broad availability, less operator dependence, faster sequences, and cost-effectiveness (101), support its value in early and accurate detection and differentiation of myocardial damage, even in preserved cardiac wall motion and cavity size. CMR in ADs provides relevant information to ensure early diagnosis and timely treatment of these high-risk patients. Despite the clear valuable role of CMR in ADs, there are still gaps in strategies and recommendations in health and intervention policy approaches for patients with ADs with CVD, requiring extensive, in-depth, multicenter clinical trials, establishing the imperative need to use CMR imaging as a non-invasive diagnostic modality of choice relevant to detect pathology in patients with multisystem autoimmune conditions.

Cardiac involvement in all mentioned ADs is there, waiting to be correctly and optimally discovered with current state-of-the-art imaging modalities. Using the proper imaging technique at the precise moment will translate into earlier and better diagnosis, adequate treatment, appropriate follow-up, and less long-term morbidity and mortality.

Currently, it is in-press and European Association of Cardiovascular Imaging (EACVI) Position Paper, entitled Role of Cardiovascular Magnetic Resonance in Autoimmune Rheumatic Diseases, where Mavrogeni et al. (268) review the promising role of CMR in the early and accurate diagnosis of the CV involvement in the AD, providing comprehensive CMR protocols and diagnostic algorithms for its use, highlighting its role to start the precise and effective specific therapeutic approach timely and not when conventional imaging modalities and the clinical manifestations show the involvement of the CV system; impacting the outcomes of these complex patients positively.

Well-designed multicenter trials to broadly confirm the findings, pathophysiological meaning, and prognostic information are needed to include CMR as part of the clinical routine in the future guidelines for the AD.

## Author Contributions

LS-G, MB, AA-D, JM-S, CS, BS, EM, and MS-L outlined, drafted, and contributed to the writing of the manuscript. All authors approved the final version of the manuscript.

## Conflict of Interest

The authors declare that the research was conducted in the absence of any commercial or financial relationships that could be construed as a potential conflict of interest.

## Publisher's Note

All claims expressed in this article are solely those of the authors and do not necessarily represent those of their affiliated organizations, or those of the publisher, the editors and the reviewers. Any product that may be evaluated in this article, or claim that may be made by its manufacturer, is not guaranteed or endorsed by the publisher.
